# RNF122 promotes glioblastoma growth via the JAK2/STAT3/c‐Myc signaling Axis

**DOI:** 10.1111/cns.70017

**Published:** 2024-09-01

**Authors:** Qingbao Xiao, Kaming Xue, Lin Li, Kai Zhu, Rong Fu, Zhiyong Xiong

**Affiliations:** ^1^ Department of Neurosurgery, Wuhan Third Hospital Tongren Hospital of Wuhan University Wuhan Hubei China; ^2^ Department of Traditional Chinese Medicine, Union Hospital, Tongji Medical College Huazhong University of Science and Technology Wuhan Hubei China; ^3^ Department of Neurosurgery, Union Hospital, Tongji Medical College Huazhong University of Science and Technology Wuhan Hubei China

**Keywords:** glioblastoma, growth, JAK/STAT/c‐Myc, RNF122

## Abstract

**Objective:**

The E3 ubiquitin ligase is well recognized as a significant contributor to glioblastoma (GBM) progression and has promise as a prospective therapeutic target. This study explores the contribution of E3 ubiquitin ligase RNF122 in the GBM progression and the related molecular mechanisms.

**Methods:**

RNF122 expression levels were evaluated using qRT‐PCR, WB, and IHC, while functional assays besides animal experiments were used to assess RNF122's effect on GBM progression. We also tested the RNF122 impact on JAK2/STAT3/c‐Myc signaling using WB.

**Results:**

RNF122 was upregulated in GBM and correlated to the advanced stage and poor clinical outcomes, representing an independent prognostic factor. Based on functional assays, RNF122 promotes GBM growth and cell cycle, which was validated further in subsequent analyses by JAK2/STAT3/c‐Myc pathway activation. Moreover, JAK2/STAT3 signaling pathway inhibitor WP1066 can weaken the effect of overexpression RNF122 on promoting GBM progression.

**Conclusion:**

Our results revealed that RNF122 caused an aggressive phenotype to GBM and was a poor prognosticator; thus, targeting RNF122 may be effectual in GBM treatment.

## Graphical abstract

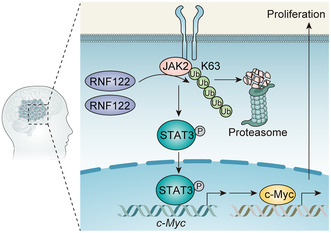

## 1 INTRODUCTION

Gliomas are extremely malignant tumors, especially glioblastoma (GBM). Despite the ongoing advancements in extensive treatments, including surgical interventions, radiotherapy, chemotherapy, and tumor treating fields technology (TTFields), the GBM survival period only increased from 14 months to about 20 months.^1^, ^2^ Therefore, an in‐depth exploration of the molecular mechanisms of glioma development and appropriate intervention is highly significant for exploring novel therapeutic targets and enhancing patient survival rates.

The E3 ubiquitin ligase, also known as E3 ubiquitin‐protein ligase, is an important protein that plays a fundamental function in the ubiquitin‐protein degradation pathway. This system is critical for regulating life processes encompassing cell cycle, signal transduction, gene expression, and apoptosis.^3^, ^4^ Kim et al. reported that the competitive interaction between the E3 ligases TRIM26 and WWP25 governs the regulation of SOX2 in GBM.^5^ Bian et al. reported that the E3 ubiquitin ligase HUWE1 inhibits the progression of GBM by the N‐Myc‐DLL1‐NOTCH1 signaling axis.^6^ In the present study, RNF122 was identified by high‐throughput protein sequencing. RNF122 refers to Ring Finger Protein 122, a gene that encodes a protein involved in the cell cycle regulation. It has been associated with various cellular processes, encompassing apoptosis (programmed cell death) and cell proliferation.^7^, ^8^ Cao et al. reported that RNF122 inhibits the antiviral type I interferon production by targeting RIG‐I CARDs to mediate RIG‐I degradation.^7^ The above literature shows that RNF122 has an essential function in biological processes. Nevertheless, the involvement of RNF122, particularly in glioma, remains unreported in the literature.

Herein, in vitro and in vivo, both loss and gain of the functional examinations showed that RNF122 enhances GBM cell growth and cell cycle. Moreover, Cignal Finder Cancer 10‐Pathway Reporter Kits were used to screen the potential signaling pathways participating in this process. Eventually, the JAK/STAT signaling axis was found to be significantly suppressed by RNF122 knocking down in LN‐229 and A‐172 cells, in contrast to the other signaling axis. Besides, based on GSEA, RNF122 showed a significant correlation to the JAK/STAT signaling pathway. Moreover, rescue experiments were conducted to validate the above finding further. Collectively, JAK2/STAT3/c‐Myc signaling axis activation by RNF122 was indicated to enhance tumor growth.

## 2 MATERIALS AND METHODS

### 2.1 Reagents and cell lines

Normal human astrocytes (HA) were cultivated in an astrocyte medium (Zeye Biotechnology, Shanghai, China). The remaining glioma cell lines are from daily storage in our laboratory and have been regularly tested for STR identification and mycoplasma. These multiple glioma cell lines U‐251, T98G, LN‐229, A‐172, and U‐87MG were cultivated according to relevant literature reports.^9^, ^10^, ^11^ JAK2/STAT3 signaling pathway inhibitor WP1066 (Selleck, S2796) was purchased from Selleck. For detailed information on antibodies, please see the Data S1.

### 2.2 Glioma samples

The normal brain tissues (NBT) were procured from individuals involved in automobile accidents who did not have glioma. Between June 2017 and January 2023, 112 glioma samples were acquired from Wuhan Union Hospital, and participant consent and ethics committee approval (UHCT‐IEC‐SOP‐016‐03‐01) were required. Aside from collecting and managing clinical samples and data according to established guidelines, no patients underwent adjuvant, neoadjuvant, or radiotherapy before surgery. Tables S1 and S2 present more detailed information.

### 2.3 Plasmid construction and lentivirus

The pLVX‐Puro‐RNF122 overexpression plasmid was constructed, followed by the transfection of LN‐229 and A‐172 cells with the respective expression plasmid. Data S1 lists the specific procedure. Briefly, LN‐229 and A‐172 cells were transfected following the protocols after their growth to 85% confluence in six‐well plates. A puromycin selection was performed on the cells after 48 h to remove uninfected cells. Based on the RNF122 sequences, siRNAs were designed, and the two siRNAs that had the most significant knock down impact were selected. For a more comprehensive examination, refer to the Data S1.

### 2.4 CCK‐8 assay

According to the protocols, cell proliferation assays were carried out with CCK‐8 (Cell Counting Kit‐8, Dojindo, Tokyo, Japan). The cells were introduced onto 96‐well plates using a fresh medium and subsequently exposed to CCK‐8 solution. Cell proliferation was determined by measuring the absorption by a microplate reader, with more available details in the previous publication.^11^, ^12^, ^13^

### 2.5 Western blotting (WB)

The tissues and cells underwent lysis and were quantified using RIPA buffer and bicinchoninic acid (BCA) (Servicebio, Wuhan, China). The lysates underwent separation through the utilization of sodium dodecyl sulfate‐polyacrylamide gel electrophoresis (SDS‐PAGE), subsequently proceeding to be moved onto a polyvinylidene fluoride (PVDF) membrane and further incubated with specific antibodies. Data S1 show detailed information on antibodies.

### 2.6 Quantitative real‐time PCR (qRT‐PCR)

Total RNA extraction, cDNA synthesis, and qRT‐PCR were performed as mentioned earlier.^10^, ^11^, ^14^ The GAPDH was employed as a standard reference gene to determine the relative amounts of mRNA expression, with more available details in the Data S1.

### 2.7 Colony formation assay

A total of 400 reconstituted cells were cultivated in a full medium for 2 weeks, describing the detailed procedures in the Data S1.

### 2.8 Cignal finder cancer 10‐pathway reporter array

A luciferase reporter was transfected into 96‐well plates containing resuspended cells to detect common cancer pathways. A luciferase reaction was performed following the protocols after incubation, with more available details in the Data S1.

### 2.9 Immunohistochemistry (IHC) and Immunofluorescence (IF)

Our previous publications provide details on immunohistochemistry (IHC) and immunofluorescence (IF).^15^, ^16^ In brief, we incubated tissues with appropriate primary and secondary antibodies after dehydrating, paraffin embedding, and sectioning them.

### 2.10 Bioinformatics analysis

The Cancer Genome Atlas Program (TCGA) (https://cancergenome.nih.gov/) was accessed to download transcript data of glioma samples and clinical information while accessing the Genotype‐Tissue Expression (GTEx, https://gtexportal.org/home/) to download the transcript data of NBT.^17^ Batch effect is a common problem in statistical analysis, especially in biological and medical research. Different experimental batches may cause non‐biological variation between data. The purpose of dealing with batch effect is to reduce or eliminate this variation so as to more accurately interpret the experimental results. The following are some common methods used by our team to deal with batch effect. (1) Normalization and standardization: During the data processing stage, normalization or standardization is used to reduce the differences between batches. For example, Z‐score standardization can be used to transform the data of each batch to a scale with the same mean and variance. (2) Batch effect correction method: (2.1) Linear regression: Use a linear regression model to remove the effect of batch as a predictor variable from the response variable. (2.2) ComBat method: This is a widely used Bayesian method suitable for adjusting for systematic differences between batches and is often used in gene expression data analysis. (2.3) Empirical Bayes method: Use information from the overall data to estimate and adjust the effects of each batch. (3) Mixed models: Using mixed effects models, batch can be included as a random effect in the model, thus accounting for the variation between batches. (4) Data integration techniques: When integrating multiple datasets, methods such as principal component analysis (PCA) and multidimensional scaling (MDS) are used to visualize and correct for batch effects. Proper handling of batch effects is a key step to ensure accurate and reliable statistical analysis results. In different situations, it may be necessary to combine one or more of the above methods.

### 2.11 Xenograft model

From Beijing Vital River Animal Technology Co. Ltd. (Beijing, China), 30 nude mice (6–8 weeks, female) were purchased. Tongji Medical College's Animal Experiments Committee approved all animal studies. A humane endpoint was established in our study following AAALAC guidelines. Following the random categorization into distinct cohorts, a cohort of female BALB/c nude mice aged 6–8 weeks underwent anesthesia with three mice in each cohort. Subsequently, a suspension of 7 μL LN‐229 and A‐172 cells (6 × 10^5^ cells) (with indicated treatment) was administered intracranially in the mouse employing a stereotaxic apparatus. Tumor size estimation was conducted utilizing the formula *V* = (*D* × *d* ^2^)/2, where *D* and *d* refer to the longest and shortest diameter, respectively.

### 2.12 Statistical analysis

First, we use normality examination to ensure that the inspection data follows a normal distribution (Gaussian distribution). The statistical analyses were performed using SPSS 22.0 software (SPSS, Inc., Chicago, IL) and GraphPad Prism (version 8.0; GraphPad Inc., La Jolla, CA, USA), reporting the data as mean ± SD. Moreover, the statistical methods employed for evaluating the significance of data include the utilization of the unpaired or paired Student's *t*‐test for two cohorts and the two‐way ANOVA, then using Dunnett's test for involving more than two cohorts. Also, the chi‐square test, Pearson's correlation, and one‐way analysis of variance were conducted. Cox regression analysis and Log‐rank test were utilized to determine survival variation and hazard ratio. *p* < 0.05 showed statistically significant. If the data do not follow a normal distribution, it may be necessary to perform a data transformation (such as a logarithmic transformation) or use nonparametric statistical methods.

## 3 RESULTS

### 3.1 RNF122 expression was increased in glioma and negatively correlated to prognosis

**FIGURE 1:**
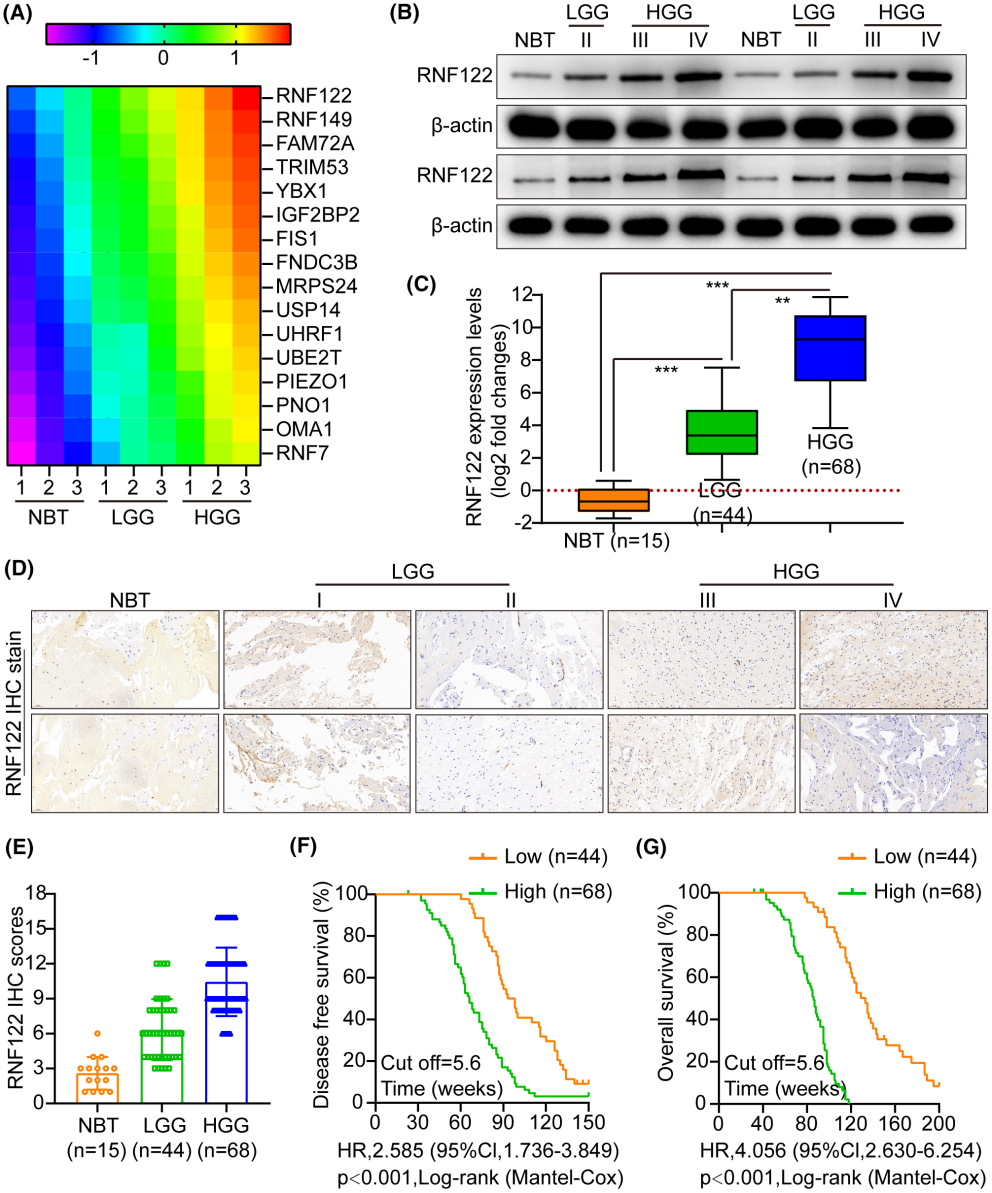
RNF122 expression was elevated in glioma and inversely connected to prognosis. (A) Heatmap displays the highest elevated genes in various cohorts. (B) WB assessed the RNF122 protein expression in glioma tissues of various grades (II–IV) and normal brain tissue (NBT). LGG: Low‐grade glioma (II), HGG: High‐grade glioma (III–IV). (C) qRT‐PCR was employed to assess RNF122 mRNA levels in glioma tissues of various grades (I–IV) and NBT. (D, E) IHC staining and scoring were employed to assess the RNF122 protein levels in glioma tissues of different grades (II–IV) and NBT. (F, G) A log‐rank test of DFS and OS was performed. The data were reported as the Mean ± SD, derived from three autonomous experiments. **p < 0.01 and ***p < 0.001.

The candidate genes contributing to tumor progression were identified using high‐throughput sequencing (NBT vs. LGG; LGG vs. HGG; NBT: NBT; LGG: low‐grade glioma; HGG: high‐grade glioma). The top 16 upregulated genes were altered more than fivefold in glioma compared to NBT (Figure 1A). RNF122 was significantly overexpressed among them. WB and qRT‐PCR analysis revealed that RNF122 was significantly overexpressed in glioma and increased with tumor grade compared to NBT, especially in HGG (Figure 1B,C). The typical IHC staining micrographs in Figure 1D indicate that RNF122 staining intensity increased significantly with tumor grade in gliomas more than in NBT. Furthermore, the IHC staining score and malignancy grade were positively correlated (Figure 1E). Kaplan–Meier analysis showed a poorer prognosis for patients with RNF122 overexpression (Figure 1F,G). Data analysis from the TCGA tumor database also demonstrated that RNF122 was overexpressed in gliomas and inversely related to patient outcomes (Figure S1A).

Herein, we conducted a receiver operating characteristic curve (ROC) analysis to compare the predictive value of RNF122‐based and WHO grade‐based, as well as the combination of both, in assessing the pathological and clinical outcomes of RNF122. The results indicate that the combination model exhibited superior predictive ability for clinical outcomes compared to the WHO grade‐based model alone (Figure S1B,C). Furthermore, the examined association between RNF122 mRNA levels and clinicopathological characteristics in 112 glioma patients (Table S1) revealed that the RNF122 mRNA expression level had a significant correlation to the tumor size (*p* < 0.001), KPS (*p* < 0.001), and WHO stage (*p* < 0.001). Based on univariate and multivariate Cox regression analyses, RNF122 mRNA expression level was correlated to WHO stage; hence, it was found to be an independent predictor of unfavorable survival outcomes in glioma patients (Table S2), indicating that RNF122 might be a potential glioma biomarker.

### 3.2 RNF122 overexpression (oeRNF122) promotes glioblastoma cell growth

**FIGURE 2:**
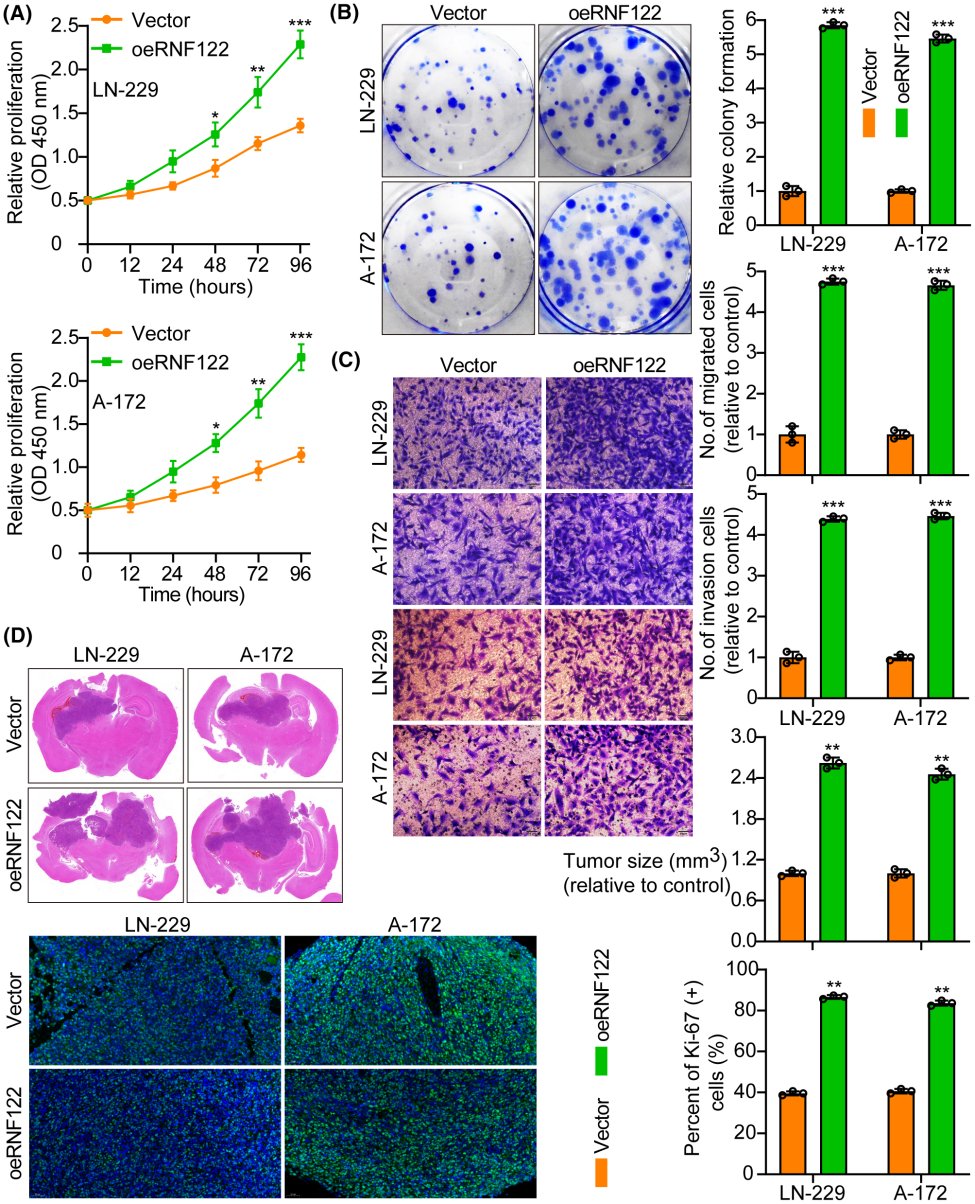
oeRNF122 promotes glioblastoma cell growth. (A) Cell growth curves were assessed employing CCK‐8 between Vector and oeRNF122. (B) oeRNF122 promoted colony formation and histogram quantification (panels). (C) Transwell migration and invasion assays show that oeRNF122 facilitated cell migration and invasion. The numbers of migrants and invading cells are displayed. Bars: 50 μm. (D) Representative frozen sections of mouse brain tissue and histogram of tumor weight and Ki‐67 staining between vector and oeRNF7. The data were reported as the Mean ± SD, derived from three autonomous experiments. *p < 0.05, **p < 0.01 and ***p < 0.001.

The first step was to measure RNF122 protein in Normal HA and five glioma cell lines (U‐251, T98G, LN‐229, A‐172, and U‐87MG) by WB. According to the results, RNF122 protein levels are greater in glioma cell lines than in HA (Figure S1D). Therefore, we selected two cell lines, LN‐229 and A‐172, with moderate expression of RNF122 protein for subsequent research. Next, overexpressed RNF122 in LN‐229 and A‐172 cells were verified using WB (Figure S1E). Based on functional tests, oeRNF122 advanced GBM cell proliferation, migration, and invasion (Figure 2A–C).

Furthermore, in vivo animal experiments again showed that oeRNF122 promoted tumor growth. Ki‐67 IF staining was utilized to assess the proliferative tumor index, indicating that proliferation in the oeRNF122 cohorts was more elevated than in the control cohorts (Figure 2D). Besides, the flow cytometric analysis revealed that the progression of the cell cycle of oeRNF122 cells was enhanced in contrast to cells transfected with the control vector (Figure S2A). The above data all suggested that oeRNF122 enhances tumor growth by promoting the cell cycle progression.

### 3.3 RNF122 knockdown suppresses glioblastoma cell growth

**FIGURE 3:**
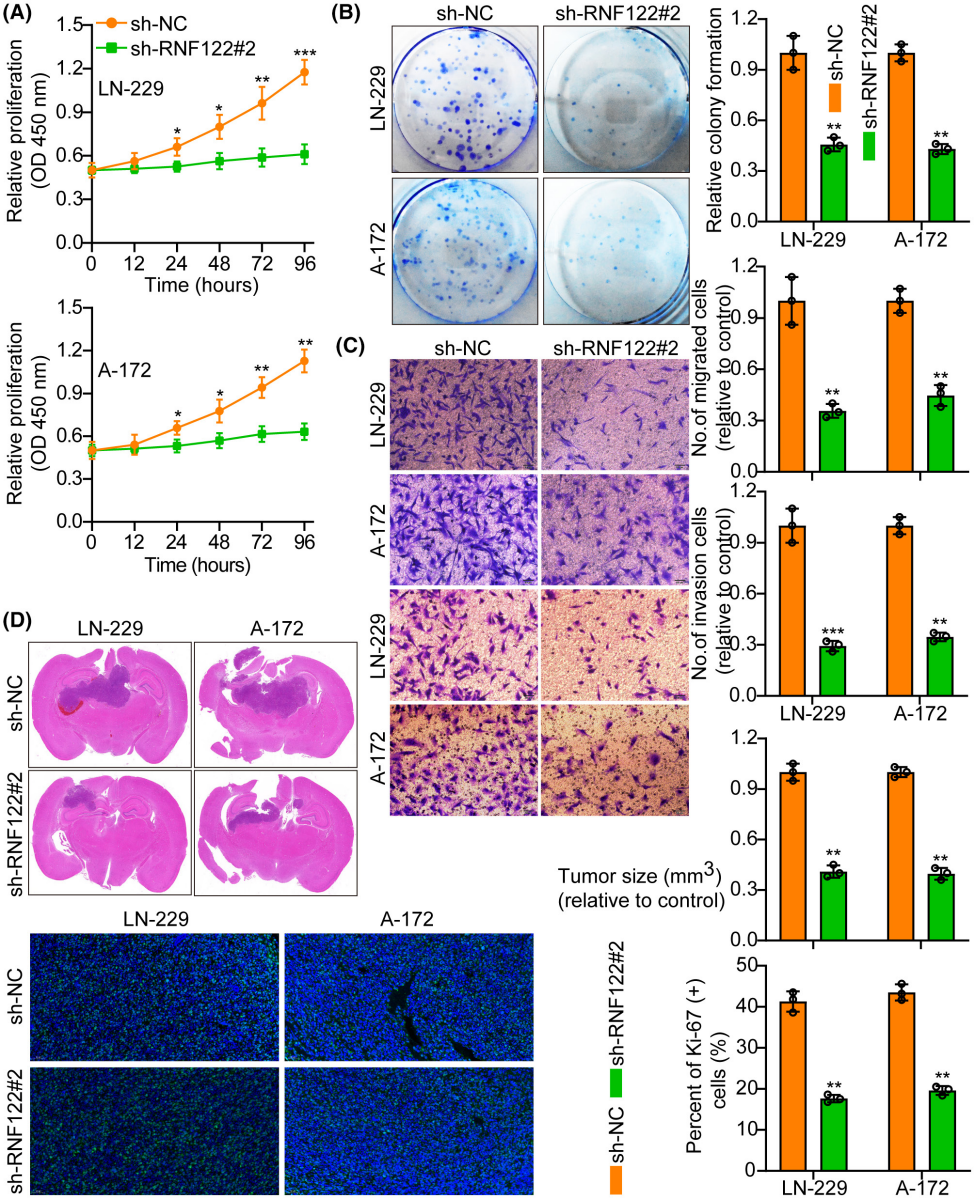
RNF122 knockdown inhibits glioblastoma cell growth. (A) Cell growth curves were determined employing CCK‐8 assay between sh‐NC and sh‐RNF122#2. (B) RNF122 knockdown suppressed colony formation and histogram quantification (panels). (C) Transwell migration and invasion assays show that the RNF122 knockdown inhibited cell migration and invasion. The quantities of migrants and invading cells are displayed. Bars: 50 μm. (D) Representative frozen sections of mouse brain tissue and histogram of tumor weight and Ki‐67 staining between sh‐NC and sh‐RNF122#2. The data were reported as the Mean ± SD, derived from three autonomous experiments. *p < 0.05, **p < 0.01 and ***p < 0.001.

RNF122 knocked down efficiency in LN‐229 and A‐172 cells, which were validated by WB (Figure S1E). The si‐RNF122#2 has the highest knockdown efficiency and will be used as a follow‐up study. Functional tests indicated that RNF122 knocking down curbed GBM cell proliferation, migration, and invasion (Figure 3A–C), which was verified further by the in vivo animal experiments. Performing Ki‐67 IF staining to assess the proliferative tumor index showed that proliferation in the RNF122 knockdown cohorts was reduced more than in the normal cohorts (Figure 3D). Additionally, it was observed through flow cytometric analysis that at the G1 phase, the cell cycle progression of cells transfected with si‐RNF122 was arrested in contrast to cells transfected with si‐NC (Figure S2B). These data suggest that RNF122 knockdown suppresses tumor growth by inducing S‐phase cell cycle arrest.

### 3.4 RNF122 activates the JAK2/STAT3/c‐Myc signaling

**FIGURE 4:**
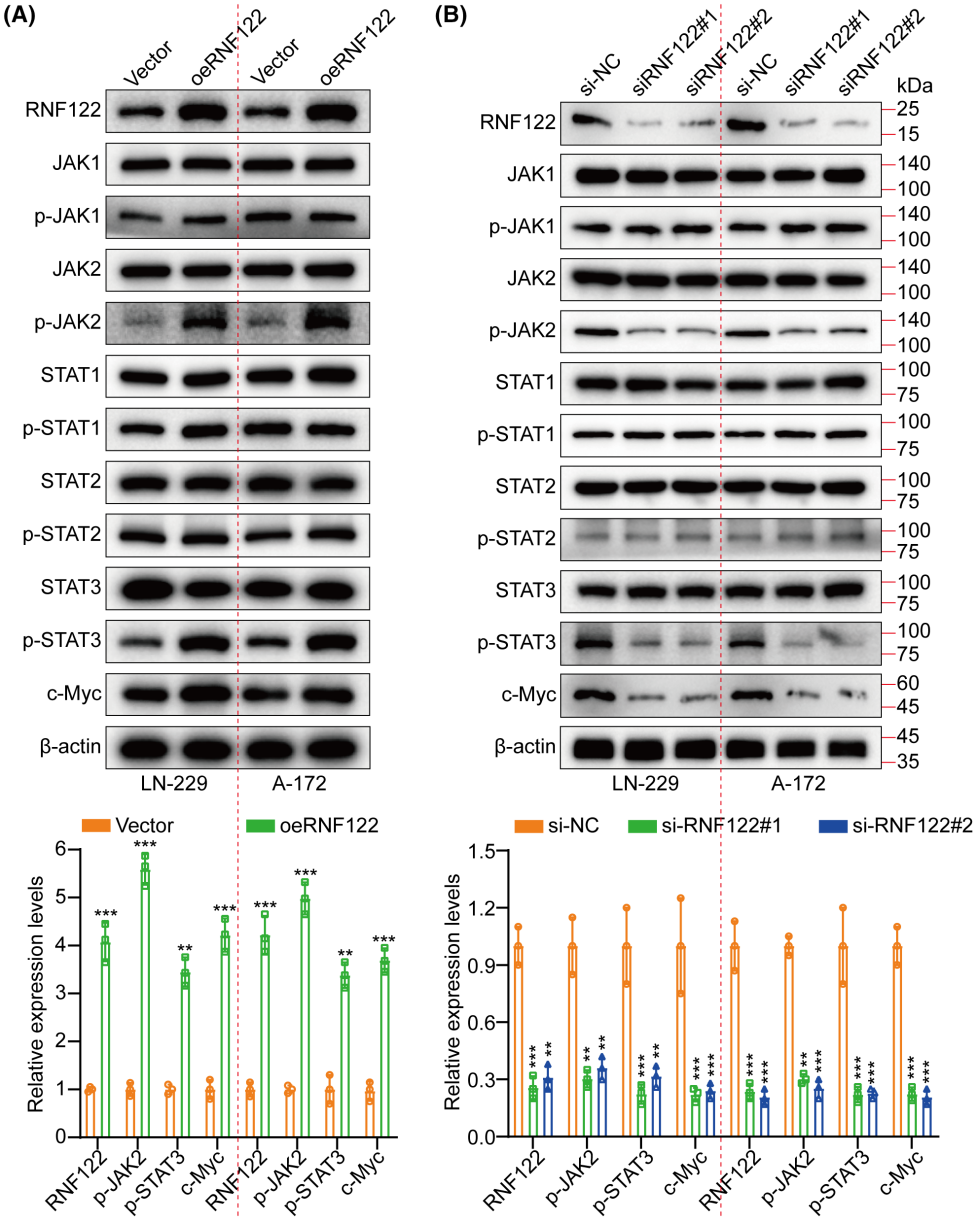
RNF122 activates the JAK2/STAT3/c‐Myc signaling. (A) JAK1, p‐JAK1, JAK2, p‐JAK2, STAT1, p‐STAT1, STAT2, p‐STAT2, STAT3, p‐STAT3, and c‐Myc expression were detected by WB between vector and oeRNF122. The downside represents the histogram. (B) Expression of JAK1, p‐JAK1, JAK2, p‐JAK2, STAT1, p‐STAT1, STAT2, p‐STAT2, STAT3, p‐STAT3, and c‐Myc were detected by WB between sh‐NC, sh‐RNF122#1, and sh‐RNF122#2. The downside represents the histogram. The data were reported as the Mean ± SD, derived from three autonomous experiments. **p < 0.01 and ***p < 0.001.

Based on the Cignal finder cancer 10‐pathway reporter array used for screening the potentially involved signaling pathways in this process, the JAK/STAT signaling axis was significantly suppressed by RNF122 knocking down in LN‐229 and A‐172 cells, in contrast to the other signaling axis (Figure S3A). Moreover, GSEA revealed that RNF122 was significantly correlated to JAK/STAT signaling (Figure S3B). Additionally, performing RNF122 knocking down and overexpressing in LN‐229 and A‐172 cells, respectively, for validating the RNF122 ability to promote tumor growth further through JAK/STAT pathway activation. WB was employed to assess the expression level of JAK1, p‐JAK1, JAK2, p‐JAK2, STAT1, p‐STAT1, STAT2, p‐STAT2, STAT3, p‐STAT3, and c‐Myc. The outcomes indicated that only p‐JAK2, p‐STAT3, and c‐Myc were downregulated by RNF122 knocking down while promoted by RNF122 overexpression (Figure 4A,B). In addition, we employed qRT‐PCR to assess the RNF122, JAK2, and STAT3 expression levels in tumor samples and performed correlation analysis. The outcomes indicated a significant positive connection between the RNF122 expression levels and the JAK2 and STAT3 (Figure S3C). Interestingly, our analysis of TCGA data also revealed a positive connection between RNF122 and JAK2, STAT3, and c‐Myc (Figure S4A). The above data all show that RNF122 activates the JAK2/STAT3/c‐Myc pathway.

### 3.5 RNF122 influences glioblastoma growth through JAK/STAT signaling activation

**FIGURE 5:**
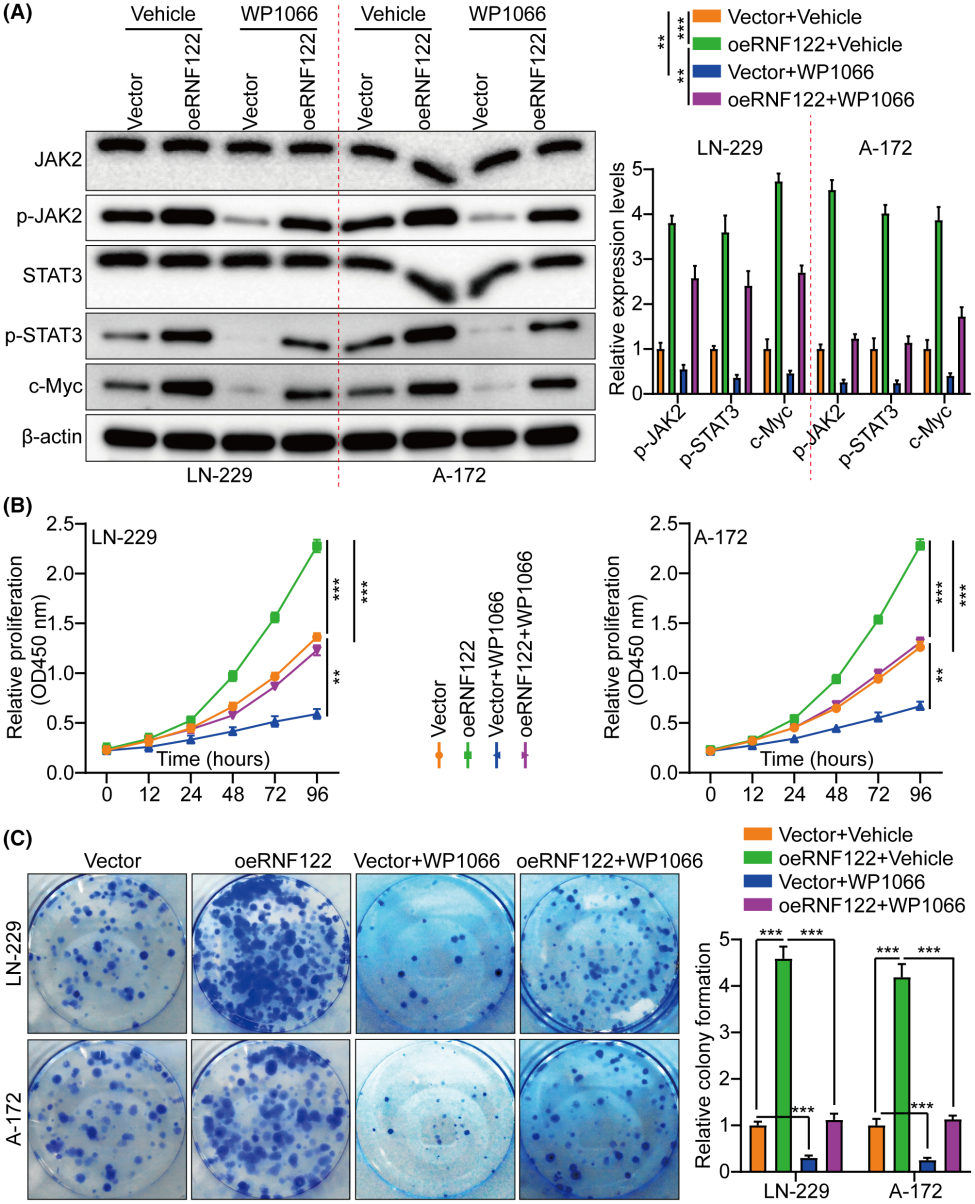
RNF122 influences glioblastoma growth by activating this JAK/STAT signaling. (A) JAK2, p‐JAK2, STAT3, p‐STAT3, and c‐Myc expression were determined through WB in different treatment cohorts. The left side represents the histogram. (B) Cell growth curves were assessed employing the CCK‐8 assay in several treatment cohorts. (C) Cell growth was assessed through colony formation assay in various treatment cohorts and histogram quantification (panels). The data were reported as the Mean ± SD, derived from three autonomous experiments. **p < 0.01 and ***p < 0.001.

**FIGURE 6:**
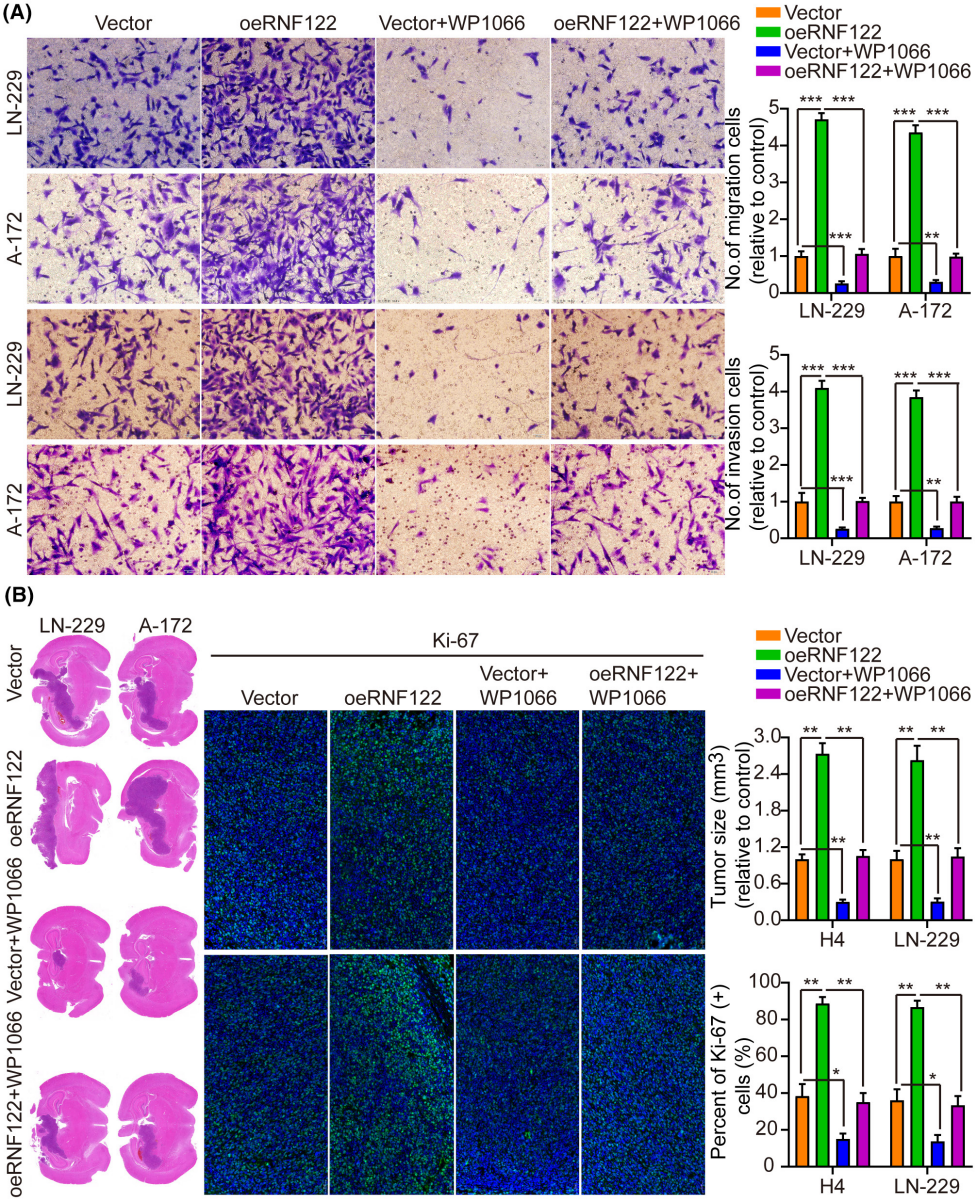
RNF122 promotes glioblastoma growth by activating this JAK/STAT signaling. (A) Transwell migration and invasion assays in various treatment cohorts and histogram quantification (panels). (B) Representative frozen sections of mouse brain tissue and histogram of tumor weight and Ki‐67 staining in various treatment cohorts. The data were reported as the Mean ± SD, derived from three autonomous experiments. *p < 0.05, **p < 0.01 and ***p < 0.001.

By not only treating LN‐229 and A‐172 cells with JAK/STAT inhibitor WP1066 (6 μM, 48 h) to further determine the RNF122 ability to promote cell proliferation through JAK/STAT activation but also by investigating RNF122 overexpression effect on proliferation, it was revealed that consistent with the earlier conclusion, RNF122‐OE induced p‐JAK2, p‐STAT3, and c‐Myc, but not JAK2 and STAT3. Nevertheless, this effect was weakened in treating LN‐229 and A‐172 cells with JAK/STAT inhibitor WP1066 (Figure 5A). Additionally, depending on the CCK‐8, colony formation, Transwell migration/invasion, and in vivo animal experiments, WP1066 led to a significant reverse of the RNF122 effect on enhancing LN‐229 and A‐172 cell proliferation, migration, and invasion (Figures 5B,C and 6A), which was parallel to the in vitro study which verified that WP1066 treatment significantly decreased tumor growth in mice with GBM intracranial orthotopic transplantation more than in the respective cohorts. The reduced Ki‐67 expression in xenograft tumors of mice treated with WP1066 implies that JAK/STAT inhibitor WP1066 could counteract the RNF122 influence on enhancing cell proliferation (Figure 6B). Collectively, RNF122 might enhance the progression of GBM by JAK/STAT signaling activation.

### 3.6 RNF122 promotes STAT3‐mediated transcription of c‐Myc

**FIGURE 7:**
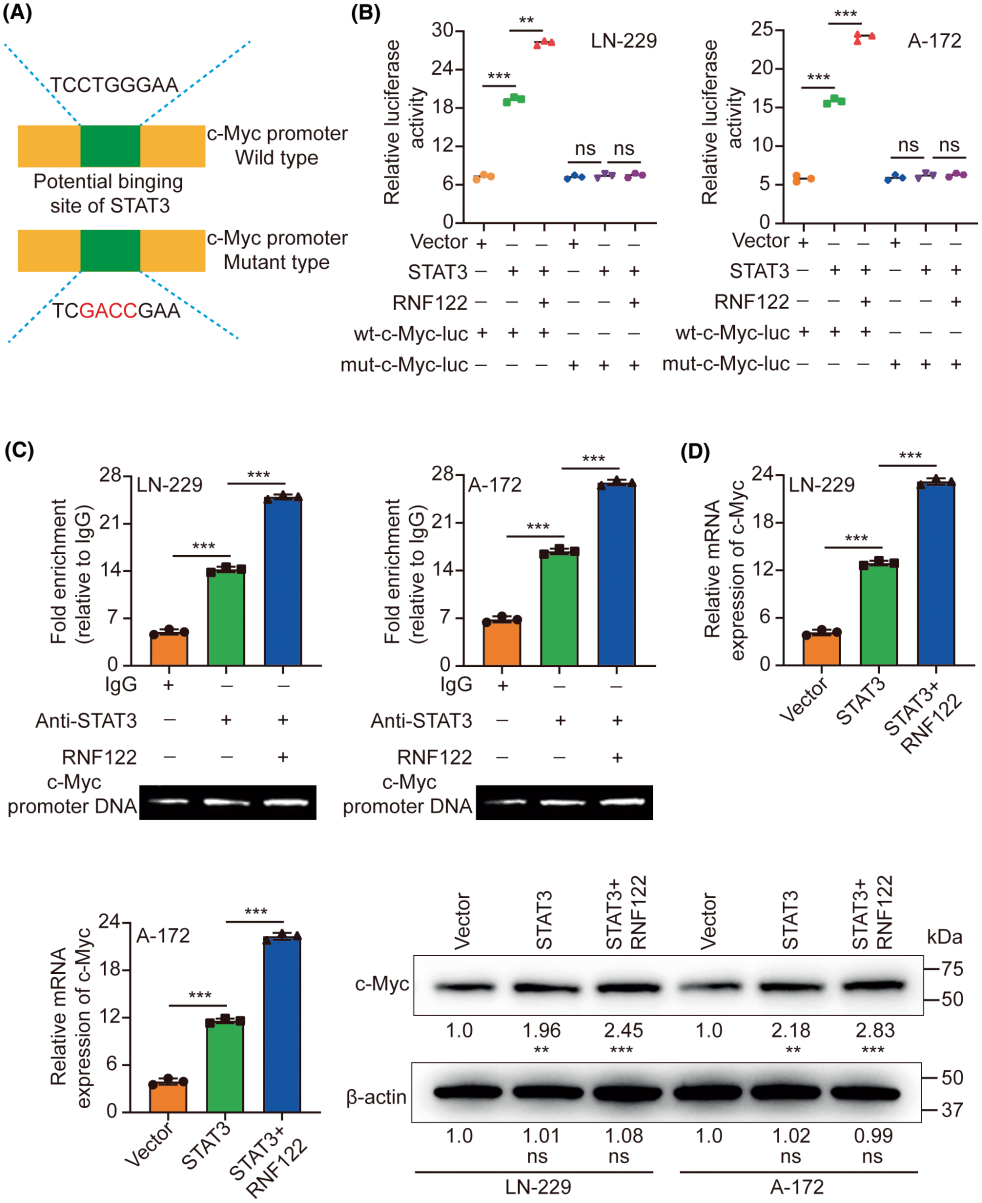
RNF122 promotes STAT3‐mediated transcription of c‐Myc. (A) The wild‐type or mutant‐type luciferase vectors were constructed according to the putative binding site of STAT3 to the c‐Myc promoter. (B) Luciferase activity was assessed in LN‐229/A‐172 cells that were transfected with luciferase vectors (either wild type or mutant type) and simultaneously co‐transfected with expression plasmids (including empty vectors, STAT3 expression plasmids, or RNF122 expression plasmids). (C) ChIP experiments were conducted to investigate the role of STAT3 in gene regulation. An internal control, IgG, was utilized to ensure the specificity of the experiment. The DNA co‐precipitated with STAT3 was then subjected to PCR amplification using primers designed for the c‐Myc promoter region. (D) The expression of c‐Myc was assessed through qRT‐PCR and WB under the ectopic expression of STAT3 or RNF122. The means ± SDs are provided (*n* = 3). **p < 0.01 and ***p < 0.001 according to two‐tailed Student *t*‐tests or one‐way ANOVA followed by Dunnett tests for multiple comparisons. Ns, no significant difference.

Transcription factors demonstrate a propensity to bind to specific DNA sequences to regulate the expression of genes. By employing the JASPAR database,^18^ the observation of STAT3 binding to the promoter of c‐Myc was determined. To demonstrate the transcriptional regulation of c‐Myc by STAT3 and the potential enhancement of this modulation by RNF122, luciferase vectors containing either the wild‐type or mutated c‐Myc promoters were generated and subsequently transfected into LN‐229/A‐172 cells (Figure 7A). The luciferase assay provided evidence that the upregulation of STAT3 led to the stimulation of WT c‐Myc promoter activity, as indicated by an increase in luciferase activity. Conversely, the overexpression did not impact mut c‐Myc promoter activity. Furthermore, RNF122 augmented the luciferase activity induced by STAT3 (Figure 7B). Moreover, the ChIP assays provided evidence of STAT3's binding to the c‐Myc promoter, with RNF122 being identified as a facilitator of this interaction (Figure 7C). The upregulation of STAT3 led to a notable increase in both c‐Myc mRNA and protein levels, subsequently facilitated by RNF122 (Figure 7D).

### 3.7 The involvement of c‐Myc in glioblastoma cell proliferation, migration, and invasion is facilitated by RNF122

**FIGURE 8:**
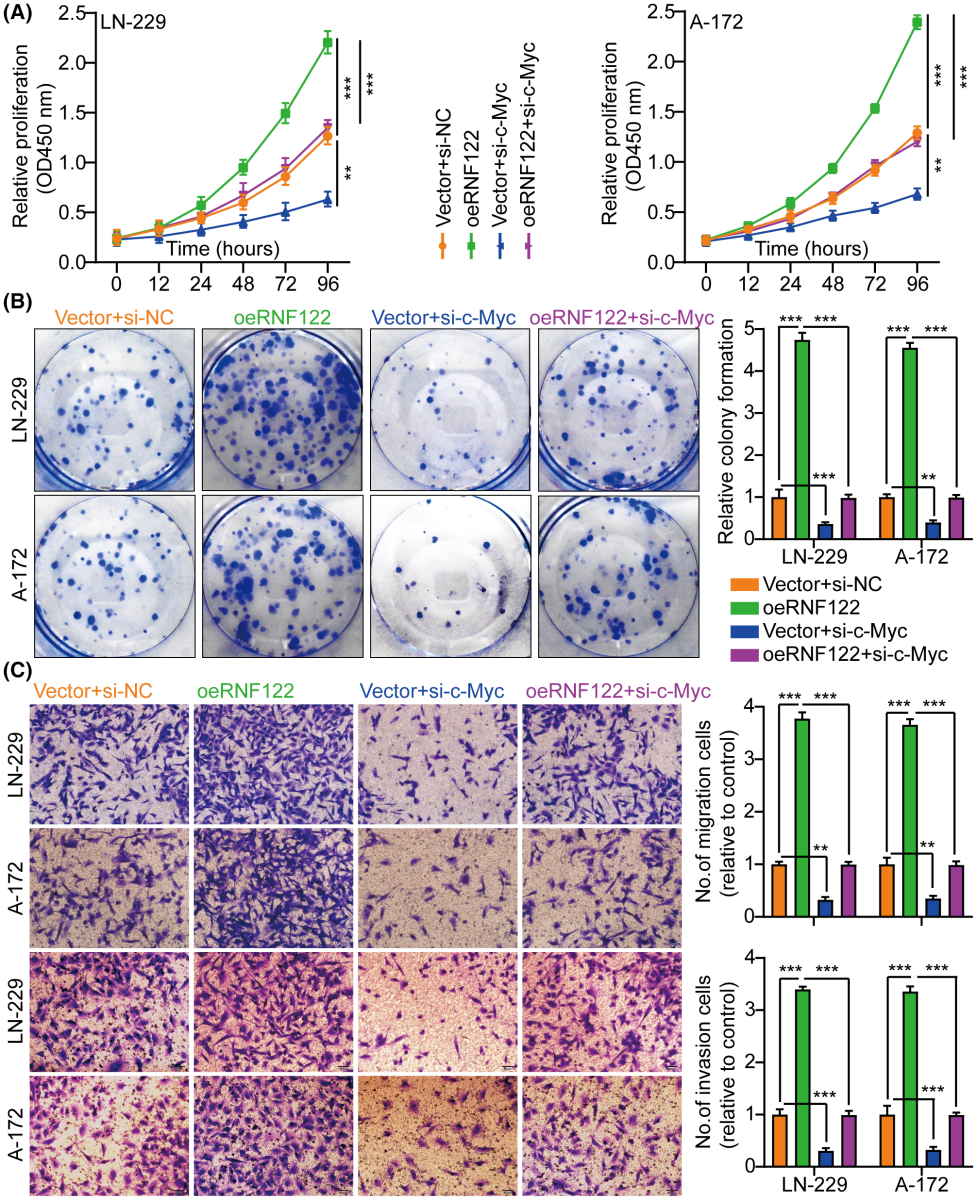
c‐Myc contributes to RNF122‐mediated glioblastoma cell proliferation, migration, and invasion. (A) Cell growth curves were assessed employing CCK‐8 assay in various treatment cohorts. (B) Cell growth was assessed employing colony formation assay in various treatment cohorts and histogram quantification (panels). (C) Transwell migration and invasion assays in various treatment cohorts and histogram quantification (panels). oeRNF122 might enhance glioblastoma cell proliferation, migration, and invasion, and the impact could be mitigated by knocking down c‐Myc. The data were reported as the Mean ± SD, derived from three autonomous experiments. **p < 0.01 and ***p < 0.001.

**FIGURE 9:**
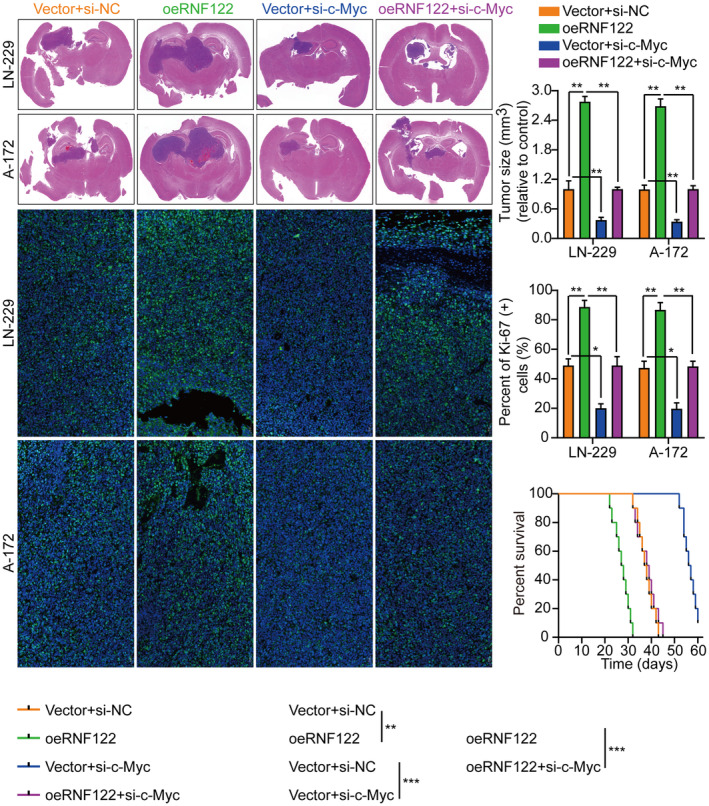
c‐Myc is involved in RNF122‐mediated glioblastoma cell growth. Representative frozen sections of mouse brain tissue and histogram of tumor weight and Ki‐67 staining in various treatment cohorts. The data were reported as the Mean ± SD, derived from three autonomous experiments. *p < 0.05, **p < 0.01 and ***p < 0.001.

The c‐Myc proteins have significant importance in the gene expression regulation of cell cycle progression, cell growth, and apoptosis, thereby being indispensable for maintaining normal cellular functionality.^11^, ^19^, ^20^ Mutations or overexpression of Myc genes has been implicated in the deregulation of cell growth and are known to have a significant function in cancer pathogenesis. The Myc gene is frequently observed to be overexpressed across diverse cancer types.^21^, ^22^, ^23^ In LN‐229 and A‐172 cells, short hairpin RNAs (siRNAs) targeting c‐Myc were utilized to investigate further its role in RNF122‐mediated GBM cell proliferation, migration, and invasion. As si‐c‐Myc#1 with the highest knockdown efficiency, it will be used as a follow‐up study (Figure S4B). Our previous data showed that c‐Myc expression was suppressed by RNF122 knocking down while promoted by RNF122 overexpression (Figure 4A,B). Besides, to verify whether RNF122 regulates c‐Myc expression further through JAK/STAT pathway activation. We overexpressed RNF122 added JAK/STAT signaling pathway inhibitors WP1066 to treat the cells and employed WB to ascertain the c‐Myc expression level. The outcomes indicated that RNF122 overexpression might increase the c‐Myc expression level, while WP1066 could weaken the ability of overexpression of RNF122 to upregulate c‐Myc protein (Figure 5A). Besides, c‐Myc silencing attenuated the promoting impacts of RNF122 overexpression on LN‐229 and A‐172 cell proliferation (Figure 8A,B), migration, and invasion (Figure 8C), which was parallel to the in vitro study, where c‐Myc silencing attenuated the promoting influences of RNF122 overexpression on LN‐229 and A‐172 cell growth in mice with GBM intracranial orthotopic transplantation tumor model more than in the corresponding cohorts. The reduced Ki‐67 expression in xenograft tumors of c‐Myc silencing cohorts also implied that c‐Myc silencing could counteract the RNF122 influence on promoting cell growth (Figure 9). Collectively, RNF122 enhances tumor development through JAK2/STAT3/c‐Myc signaling axis activation.

## 4 DISCUSSION

**FIGURE 10:**
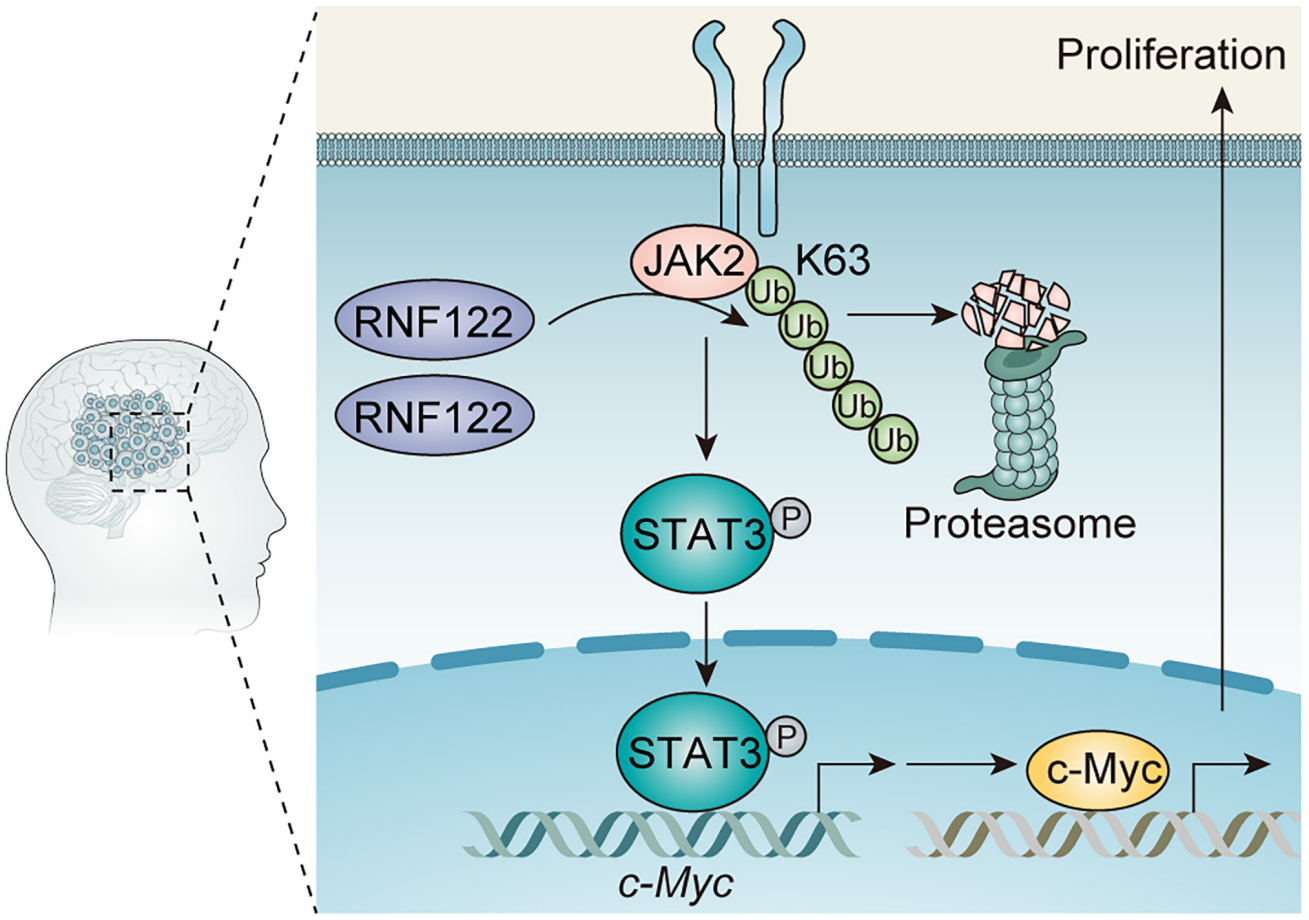
Schematic diagram of the mechanism. RNF122 catalyzes the non‐degradative ubiquitination modification of K63 of JAK2 and further promotes the phosphorylation activation of JAK2. Phosphorylated JAK2 then activates its downstream protein STAT3. The phosphorylated STAT3 enters the nucleus and promotes the transcriptional activation and protein expression level of c‐Myc. As a common oncogenic protein, c‐Myc can further promote the malignant progression of glioblastoma.

Although RNF122 dysregulation and its biological role in several disorders have been reported before,^7^, ^8^, ^24^ its possible role in glioma remains unreported. Herein, the RNF122 clinical relevance as a prognostic marker, as well as its biological role in glioma, was reported, revealing that the overexpressed RNF122 in human glioma tissue and cell lines improved growth of glioma cells, showing that RNF122 is involved in glioma. Mechanistically, RNF122 may enhance tumor progression through JAK2/STAT3/c‐Myc signaling pathway activation (Figure 10).

The specific function of RNF122 may vary in different cellular contexts. E3 ligases, such as RNF122, frequently mediate diverse cellular mechanisms, encompassing cell cycle progression, DNA repair, and cellular stress response. Wang et al.^7^ suggested that RNF122 roles as an inhibitor of the type I interferon production, an antiviral response, through its ability to target the RIG‐I CARDs and facilitate the RIG‐I degradation. Sun et al.^8^ reported that the orchestration of porcine E3 ubiquitin ligase RNF122 by PRRSV nonstructural proteins facilitates the proliferation of PRRSV. Ribasés et al.^24^ reported that the findings of a Gene‐wide Association Study have unveiled that RNF122 Ubiquitin Ligase represents a recently discovered susceptibility gene for Attention Deficit Hyperactivity Disorder. Nevertheless, in glioma, neither the function nor the mechanism of RNF122 was clarified. Herein, RNF122 was overexpressed in glioma, and the RNF122 overexpression was discovered to be associated with a poor outcome and may serve as an autonomous prognostic indicator for glioma. RNF122 promoted tumor cell growth based on the loss/gain of function. Moreover, the Cignal finder cancer 10‐pathway reporter array revealed that the JAK/STAT signaling axis showed a significant inhibition due to RNF122 knocking down in LN‐229 and A‐172 cells, in contrast to the other signaling axis. Additionally, GSEA results revealed that the JAK/STAT signal path might be associated with RNF122 function in the tumor, which was validated further with WB and rescue experiments by the widely used JAK/STAT inhibitor WP1066.^25^, ^26^ The JAK/STAT signaling pathway, renowned for its high activity levels in tumors, has been documented to have a significant function in developing malignancies.^27^, ^28^ Wang et al.^29^ reported that IGF2BP3 has a crucial function in promoting cell proliferation and tumorigenesis in human bladder cancer by modulating the JAK/STAT signaling pathway. Yang et al.^30^ reported that ADAMTS10 effectively suppresses aggressiveness in gastric cancer by modulating the JAK/STAT/c‐MYC pathway and inducing macrophage reprogramming, thereby establishing a favorable microenvironment that counteracts malignancy. Chen et al.^31^ reported that the inhibition of ATM has the potential to achieve the reversal of epithelial‐mesenchymal transition (EMT) and the reduction in metastatic ability of cisplatin‐resistant lung cancer cells, which operates via the JAK/STAT3/PD‐L1 pathway. Yu et al.^32^ reported the criticality of fatty acid β‐oxidation, regulated by JAK/STAT3, in breast cancer stem cell self‐renewal and chemoresistance. The reports above, in addition to our research, provide evidence suggesting that the JAK/STAT signal pathway leads to the malignant progression of tumors.

Due to their importance in cancer biology, c‐Myc proteins are extensively studied in research laboratories. Understanding the molecular mechanisms controlled by c‐Myc is crucial for developing targeted cancer therapies.^21^, ^33^ Gao et al.^34^ discovered the impact of c‐Myc‐induced alterations in cancer cell energy metabolism and its potential for therapeutic interventions. Liu et al.^35^ discovered that NCAPD3 amplifies the Warburg effect via the activation of c‐Myc and E2F1, thereby facilitating the initiation and advancement of colorectal cancer. Dalton et al.^36^ reported a convergence between the cell cycle and c‐Myc regarding the regulatory mechanisms that govern pluripotency and reprogramming. Zhang et al.^37^ reported that HOXB9 achieves the suppression of pancreatic cancer cell proliferation via the DNMT1/RBL2/c‐Myc axis, which effectively blocks cell cycle progression. Li et al.^11^ reported that glioma progression is facilitated by regulating the MZF1/c‐Myc/HIF1‐α axis through the presence of Linc01060 in exosomes derived from hypoxic glioma stem cells. Interestingly, our results supported the idea that c‐Myc could promote glioma growth.

As an E3 ubiquitin ligase, how does RNF122 activate JAK2/STAT3 pathway? To further elucidate this issue, we conducted a series of related experiments. It is well known that after monoubiquitination of a substrate, ubiquitin can bind to other ubiquitins through any of the seven lysine (Lys) residues (Lys6, Lys11, Lys27, Lys29, Lys33, Lys48, and Lys63) or Met1 to form polyubiquitination. Lys48‐linked ubiquitination mediates substrate degradation via the proteasome pathway, accounting for half of all ubiquitination forms. The second is K63 (Lys63)‐linked ubiquitination, which promotes the degradation of protein substrates and related cellular contents (such as damaged mitochondria and invading pathogens) via the autophagy pathway, or regulates the non‐degradation processes of substrate molecules, including protein trafficking, DNA repair, protein kinase activation, and molecular interactions. For example, the E3 enzyme TRAF6 mediates K63 ubiquitination of AKT, promoting its recruitment to the cell membrane and phosphorylation activation.^38^ TRIM31 catalyzes K63‐linked polyubiquitination of Lys10, Lys311, and Lys461 on the signaling adaptor molecule MAVS, leading to the formation of prion‐like aggregates of MAVS and the activation of innate antiviral immune responses.^39^ Growth factors promote RICTOR‐mTOR binding, mTORC2 complex assembly, and signal transduction by regulating the removal of K63 ubiquitin modification on RICTOR by ubiquitin‐specific protease 9X (USP9X).^40^

Herein, when we explored the regulation of RNF122 on JAK2, we unexpectedly found that overexpression or silencing of RNF122 did not change the expression levels of JAK2 protein and mRNA (Figure S5A,B), and the actinomycin (CHX) experiment confirmed that overexpression of RNF122 could not promote JAK2 degradation (Figure S5C). Co‐IP experiments further showed that RNF122 promoted the increase of total ubiquitination modification level of JAK2 (Figure S5D), but upregulated K63 rather than K48‐linked ubiquitination (Figure S5E). Because we found that RNF122 catalyzes the non‐degradative ubiquitination modification of K63 of JAK2, we speculate that RNF122 may regulate the activity of JAK2, that is, promote the phosphorylation activation of JAK2.

The specific role and biological significance of RNF122 (RING finger protein 122) in GBM have not been fully elucidated. Here we only reveal part of its function. RNF122 affects the activity of JAK2 protein kinase in GBM through its E3 ubiquitin ligase activity. However, as a member of the RING finger protein family, RNF122 is likely to play a more important role in the development and treatment of GBM. For example, in GBM, RNF122 may affect PI3K/Akt, MAPK, or other pathways associated with tumor proliferation and survival. In addition, RNF122 may also be involved in regulating the expression of specific genes, thereby affecting cell behavior, including proliferation, invasion, and drug resistance. In this study, we found that RNF122 is highly expressed in GBM and is closely related to the progression or prognosis of the disease, so it may become a new therapeutic target. Develop or screen for compounds that can directly inhibit the activity of RNF122, which can be used alone or in combination with other antitumor drugs. Combining RNF122 inhibitors with existing treatments such as radiation therapy, chemotherapy, or other targeted therapies such as EGFR inhibitors may enhance therapeutic efficacy, especially when there is synergistic effect on specific signaling pathways. Viral vectors are used to directly interfere with the expression of RNF122, reducing its level in tumor cells and thus inhibiting tumor growth.

## 5 CONCLUSION

In summary, our data indicated that RNF122 activated the JAK2/STAT3/c‐Myc axis to facilitate GBM cell proliferation and cell cycle and RNF122 activated JAK2/STAT3 signaling, which promoted c‐Myc protein expression and promoted GBM progression. In conclusion, c‐Myc is regulated by RNF122 through JAK2/STAT3 pathway activation, and our results provide a model via which RNF122 accelerates GBM progression. As a result, we suggest and elucidate that RNF122 enhances GBM progression via the JAK2/STAT3/c‐Myc signal pathway and that these molecules may be promising novel targets.

## AUTHOR CONTRIBUTIONS

Q. Xiao and Z. Xiong designed the investigation and wrote the manuscript; L. Li, K. Zhu, R. Fu, and Z. Xiong collected clinic samples; Q. Xiao, K. Xue, R. Fu, and Z. Xiong experimented and analyzed the data.

## CONFLICT OF INTEREST STATEMENT

The authors assert that they do not possess any conflicting interests.

## Supporting information


Data S1.

## Data Availability

The data that support the findings of this study are available from the corresponding author upon reasonable request.
