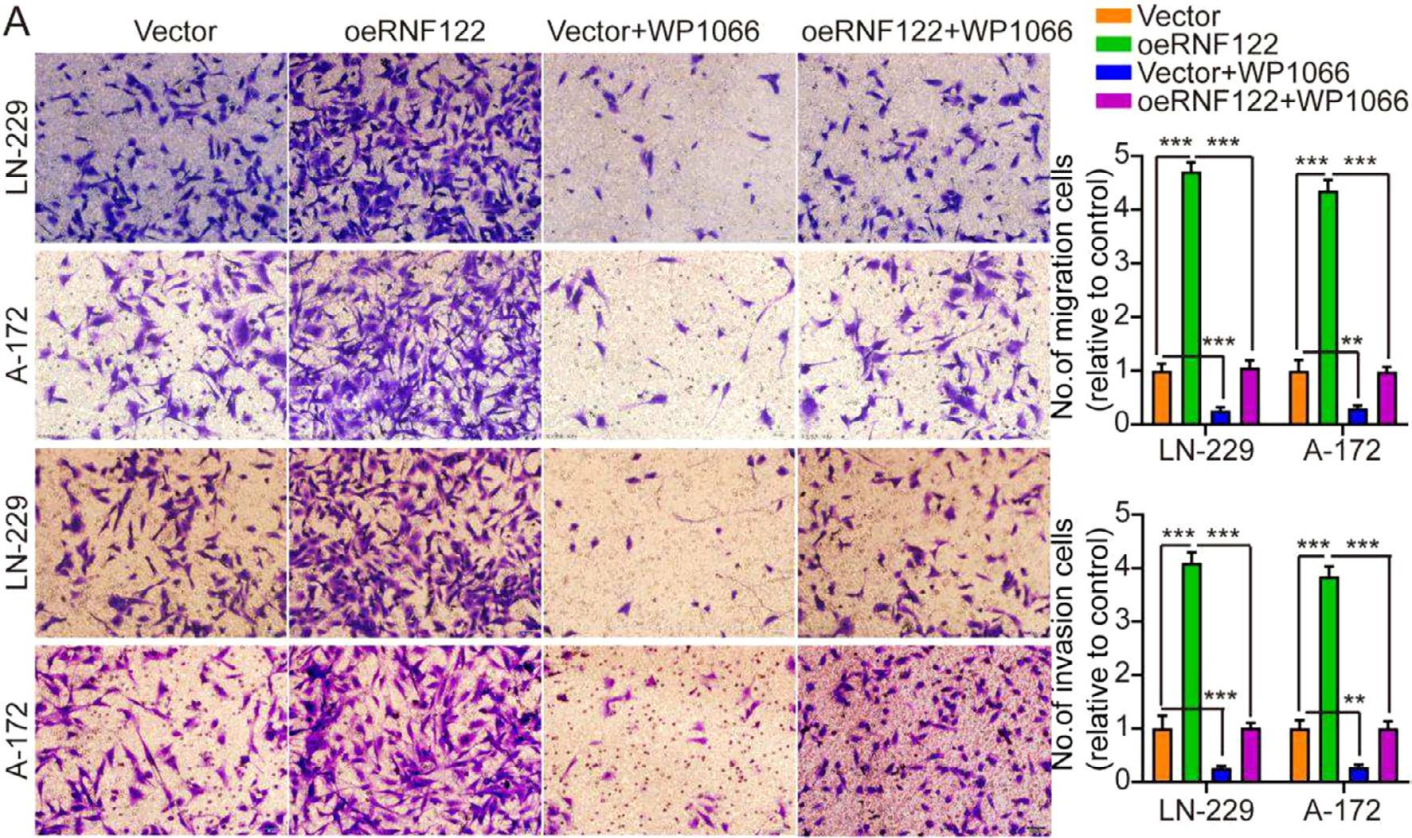# Correction to “RNF122 Promotes Glioblastoma Growth via the JAK2/STAT3/c‐Myc Signaling Axis”

**DOI:** 10.1111/cns.70317

**Published:** 2025-03-12

Xiao Q, Xue K, Li L, Zhu K, Fu R, Xiong Z, RNF122 Promotes Glioblastoma Growth via the JAK2/STAT3/c‐Myc Signaling Axis, CNS Neuroscience & Therapeutics 30, (2024): e70017 10.1111/cns.70017.

In the original version of our article, there was an error in Figure 6A. Specifically, the representative image of A‐172 (oeRNF122 + WP1066) cells in Figure 6A is incorrect. The correct image is provided below. This correction will not affect the results and conclusions.

We sincerely apologize for this error in the article and for any inconvenience caused.