# Supplementary material for: RNF122 promotes glioblastoma growth via the JAK2/STAT3/c‐Myc signaling Axis
Source: CNS Neurosci Ther. 2024 Sep 1;30(9):e70017. doi: 10.1111/cns.70017 (PMC11366496; doi:10.1111/cns.70017)
Supplement: Supplementary file 1 — Data S1. [file CNS-30-e70017-s001.zip › cns70017-sup-0001-Supinfo01.docx]

**RNF122 Promotes Glioma Growth via the JAK2/STAT3/c-Myc Signaling Axis**

Qingbao Xiao^1#^, Kaming Xue^2#^, Lin Li^3^, Kai Zhu^3^, Rong Fu^3*^, Zhiyong Xiong^3*^

**Materials and Methods**

**Reagents and cell lines**

Normal human astrocytes (HA) and multiple glioma cell lines U-251, T98G, LN-229, A-172, and U-87MG were cultivated according to the supplier’s cultivation suggestion. WP1066 was purchased from Selleck (Selleck, S2796). Primary antibodies were utilized for detecting RNF122 (Ag22511; Proteintech), JAK1 (ab133666; Abcam), p-JAK1 (ab138005; Abcam), JAK2 (ab108596; Abcam), p-JAK2 (ab32101; Abcam), STAT1 (ab234400; Abcam), p-STAT1 (ab109461; Abcam), STAT2 (ab32367; Abcam), p-STAT2 (ab191601; Abcam),STAT3 (ab68153; Abcam), p-STAT3 (ab267373; Abcam), c-Myc (ab32072; Abcam), and β-actin (#4967; [Cell Signaling Technology](http://www.baidu.com/link?url=RmrODVtP-QELNFdwcO1OKsapYtf8n2VnECvVHSXBpXWQnW6HMgckJvLz3pHEbF64)).

**Plasmids, small interfering RNA (siRNA), and transfection**

The LN-229 and A-172 cells were transfected with the shRNAs targeting RNF122 and c-Myc, and RNF122 using Lipofectamine 3000 (Invitrogen). We constructed the RNF122 pcDNA3.1 (oeRNF122 (overexpression)) inserting the respective genes into the pcDNA3.1 vector (Invitrogen). Empty pcDNA3.1 vector (Vector) was used as control. All the siRNAs and shRNF122/c-Myc and shNC lentiviral vectors were obtained from GeneChem Co., Ltd (Shanghai, China). Supplementary Table 3 lists the sequences of relative siRNAs and shRNAs.

**Real-time quantitative RT-PCR (qRT-PCR)**

Total RNA was isolated from the cells and tissues using TRIzol and TRIzol LS reagents (Life Technologies). The miRNAs were reverse transcribed using the Mir-X™ miRNA First-Strand Synthesis Kit (Clontech, Mountain View, CA, USA). Real-time PCR was performed using the SYBR Green PCR Master Mix (Takara, Shiga, Japan) and the primers listed in Supplementary Table 4. The mRNA levels were measured with the 7500 Fast Real-Time PCR Systems (Applied Biosystems, Foster City, CA, USA). β-actin was used as internal control.

**Colony formation assay**

Four hundred cancer cells were seeded in a 6-cm plate using two ml of complete medium. Over a period of two or three weeks, the medium was changed twice a week at 37°C with 5% CO2 . Colonies were then washed twice with PBS and fixed with paraformaldehyde at 4%. Colonies were stained with crystal violet (Servicebio, Hubei, China). Counting colonies under an inverted microscope was performed at the end of the experiment. At least three independent experiments were conducted.

**Cignal finder cancer 10-pathway reporter array**

All operations were performed according to the reagent manufacturer's instructions. Cells were resuspended and plated into 96-well plates along with luciferase reporters targeted at common cancer pathways. Luciferase activity was then detected after the cells were incubated.

**Supplementary Tables 1:** Correlation of the expression levels of RNF122 in glioma tissues with clinicopathologic features.

| Features | No. | RNF122 | | P-value |
| --- | --- | --- | --- | --- |
|  |  | Low | High |  |
| Age(years) |  | | | |
| <50 | 48 (42.9%) | 25 (22.3%) | 23 (20.5%) | 0.301 |
| >=50 | 64 (57.1%) | 27 (24.1%) | 37 (33.1%) |  |
| Gender |  | | | |
| Male | 60 (53.6%) | 32 (28.6%) | 28 (25.0%) | 0.119 |
| Female | 52 (46.4%) | 20 (17.8%) | 32 (28.6%) |  |
| Tumor size, cm |  | | | |
| <2 | 65 (58.0%) | 40 (35.7%) | 25 (22.3%) | P<0.001*** |
| >=2 | 47 (42.0%) | 12 (10.7%) | 35 (31.3%) |  |
| Tumor location |  | | | |
| Supratentorial | 46 (41.1%) | 21 (18.8%) | 25 (22.3%) | 0.878 |
| Subtentorial | 66 (58.9%) | 31 (27.6%) | 35 (31.3%) |  |
| Karnofsky performance scale |  | | | |
| <90 | 62 (55.4%) | 20 (17.9%) | 42 (37.5%) | P<0.001*** |
| >=90 | 50 (44.6%) | 32 (28.5%) | 18 (16.1%) |  |
| WHO grade |  | | | |
| Low-grade(Ⅰ+Ⅱ) | 44 (39.3%) | 30 (26.8%) | 14 (12.5%) | P<0.001*** |
| High-grade(Ⅲ+Ⅳ) | 68 (60.7%) | 22 (19.6%) | 46 (41.1%) |  |
| Tumor recurrence |  | | | |
| No | 58 (51.8%) | 36 (32.2%) | 22 (19.6%) | P<0.001*** |
| Yes | 54 (48.2%) | 16 (14.3%) | 38 (33.9%) |  |

statistics based on available data. ***<0.001. RNF122 high expression: score 8-16; low expression: score 0-7.

**Supplementary Tables 2:** Univariate and multivariate for clinicopathological features associated with various prognostic parameters of 112 glioma patients by Cox-regression analysis.

| Variables | Univariate analysis | | Multivariate analysis | |
| --- | --- | --- | --- | --- |
|  | HR (95%CI) | P-value | HR (95%CI) | P-value |
| WHO grade (I+II vs III+IV) | 1.952 (1.164-3.285) | 0.008** | 1.495 (1.038-2.849) | 0.016* |
| Tumor size (≥2cm vs ＜2cm) | 1.237  (0.895-2.628) | 0.037* | 1.036 (0.827-2.462) | 0.072 |
| Tumor recurrence (Yes vs No) | 2.649 (1.851-4.946) | p<0.001*** | 1.128 (0.952-2.793) | 0.054 |
| RNF122 (Low vs High) | 1.359  (1.145-3.173) | 0.027* | 1.096 (0.875-2.853) | 0.068 |

| **Gene** | **Primer** | **Sequence(5′-3′)** |
| --- | --- | --- |
| si-RNF122#1 | forward | CGGGCTCTTACCTCTCTACAA |
| si-RNF122#2 | forward | CCTTATCTTCTGCTGCTATTT |
| si-c-Myc#1 | forward | CCTGTGCCACTAAACTACATT |
| si-c-Myc#2 | forward | CGAGGACATCTGGAAGAAATT |

**Supplementary Tables 3:**

**Supplementary Tables 4:**

| **Gene** | **Primer** | **Sequence(5′-3′)** |
| --- | --- | --- |
| RNF122 | forward | ATTCCAGTGGTGTAACGGGTG |
|  | reverse | CCTGTGCCGAAGATGACCATA |
| c-Myc | forward | GGCTCCTGGCAAAAGGTCA |
|  | reverse | CTGCGTAGTTGTGCTGATGT |
| GAPDH | forward | GAGTCAACGGATTTGGTCGT |
|  | reverse | TTGATTTTGGAGGGATCTCG |

**Supplementary figure legends**

**
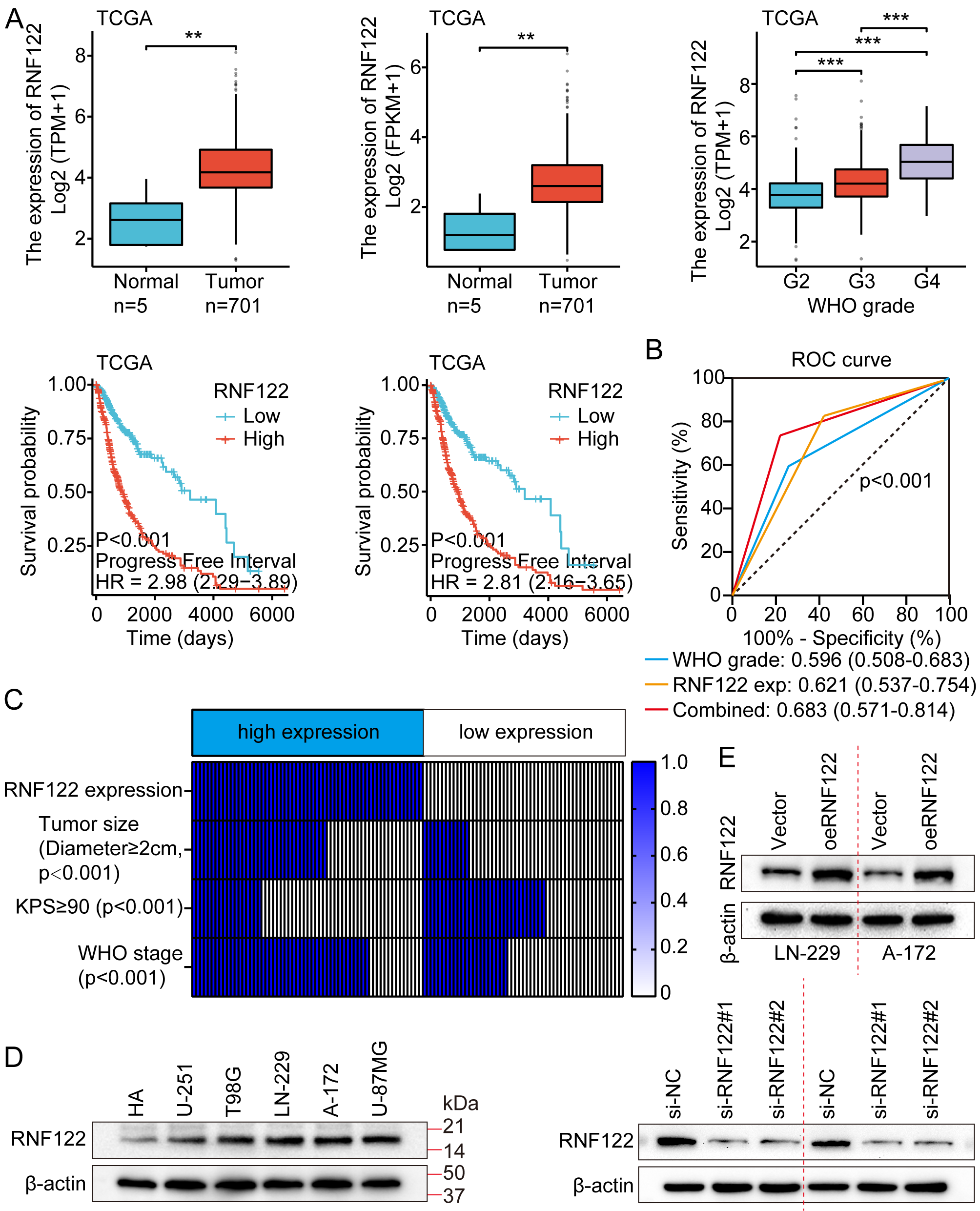
**

**Figure S1 A** TCGA database results show that RNF122 is highly expressed in tumor tissues and is inversely related to patient prognosis. **B** ROC analysis of RNF122-based, WHO-based and the combination model in predicting clinical outcome. **C** The heatmap illustrates the association of different clinical characters with RNF122 high and low-expression tumors. **D** WB to detect the expression levels of RNF122 between HA, U-251, T98G, LN-229, A-172 and U-87MG cells. **E** Validation of overexpression and knockdown efficiency of RNF122 by WB. Data shown are mean ± SD (n = 3). (**P < 0.01 and ***P < 0.001).

**
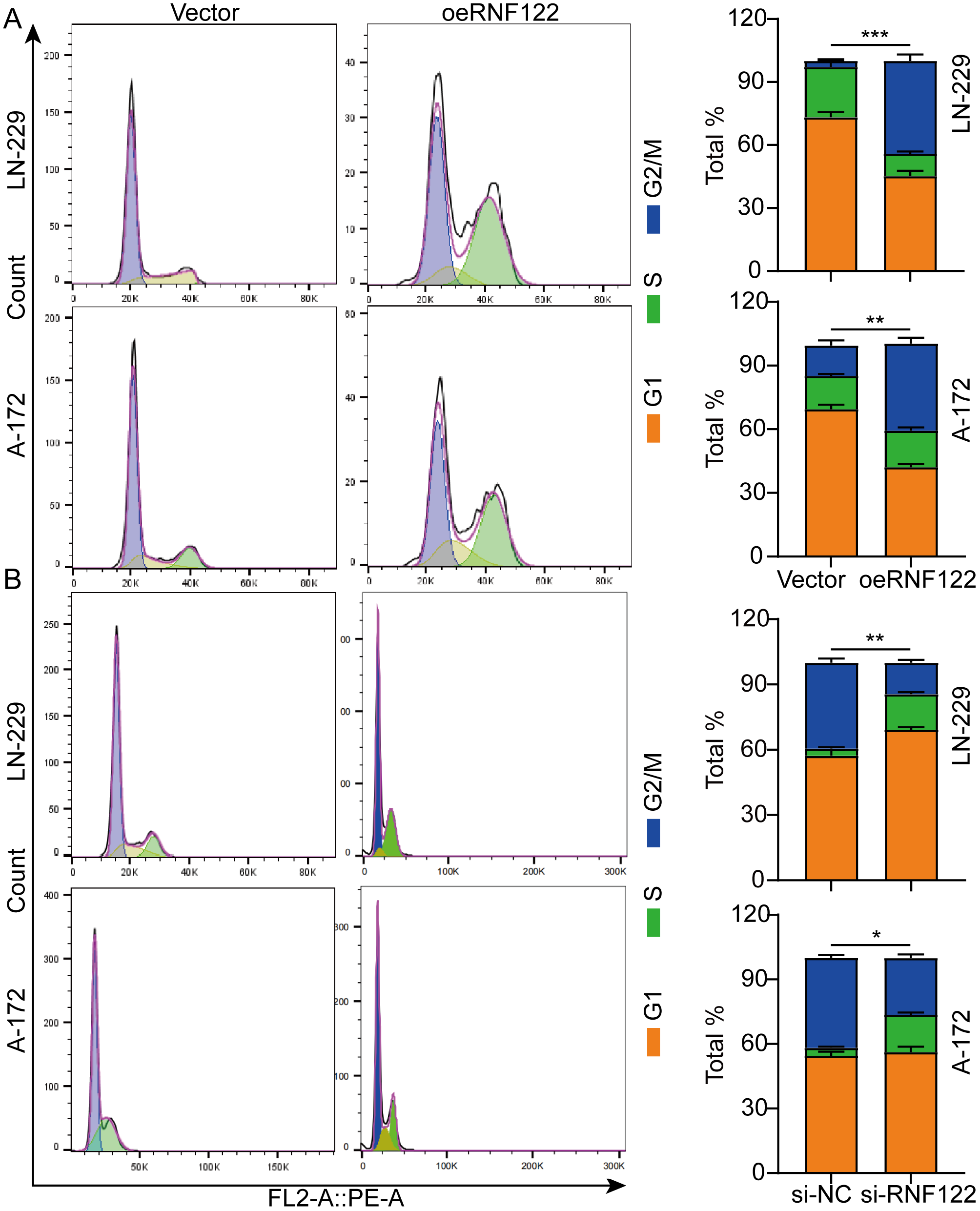
**

**Figure S2 A** The flow cytometric analysis revealed that the progression of the cell cycle of oeRNF122 cells was enhanced in contrast to cells transfected with the control vector. **B** It was observed through flow cytometric analysis that at the G1 phase, the cell cycle progression of cells transfected with si-RNF122 was arrested in contrast to cells transfected with si-NC.

**
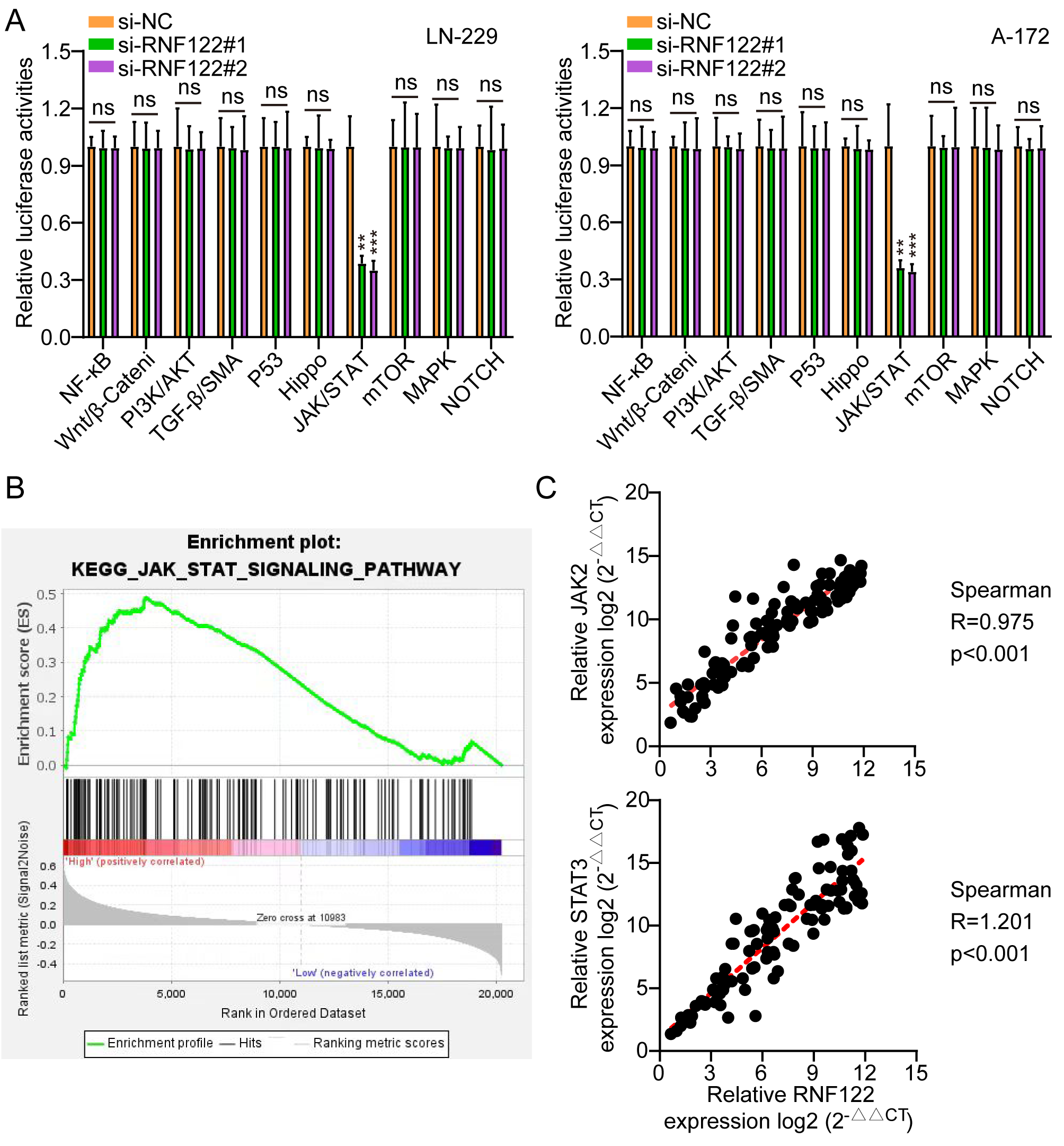
**

**Figure S3 A** Luciferase reporter gene assay results between si-NC, si-RNF122#1 and si-RNF122#2. **B** GSEA was performed using the GSEA software. **C** Our results show that RNF122 is positively correlated with the expression of JAK2 and STAT3 by PCR. The means ± SDs are provided (n=3). **P < 0.01 and ***P < 0.001 according to two-tailed Student t tests or one-way ANOVA followed by Dunnett tests for multiple comparisons. ns, no significant difference.


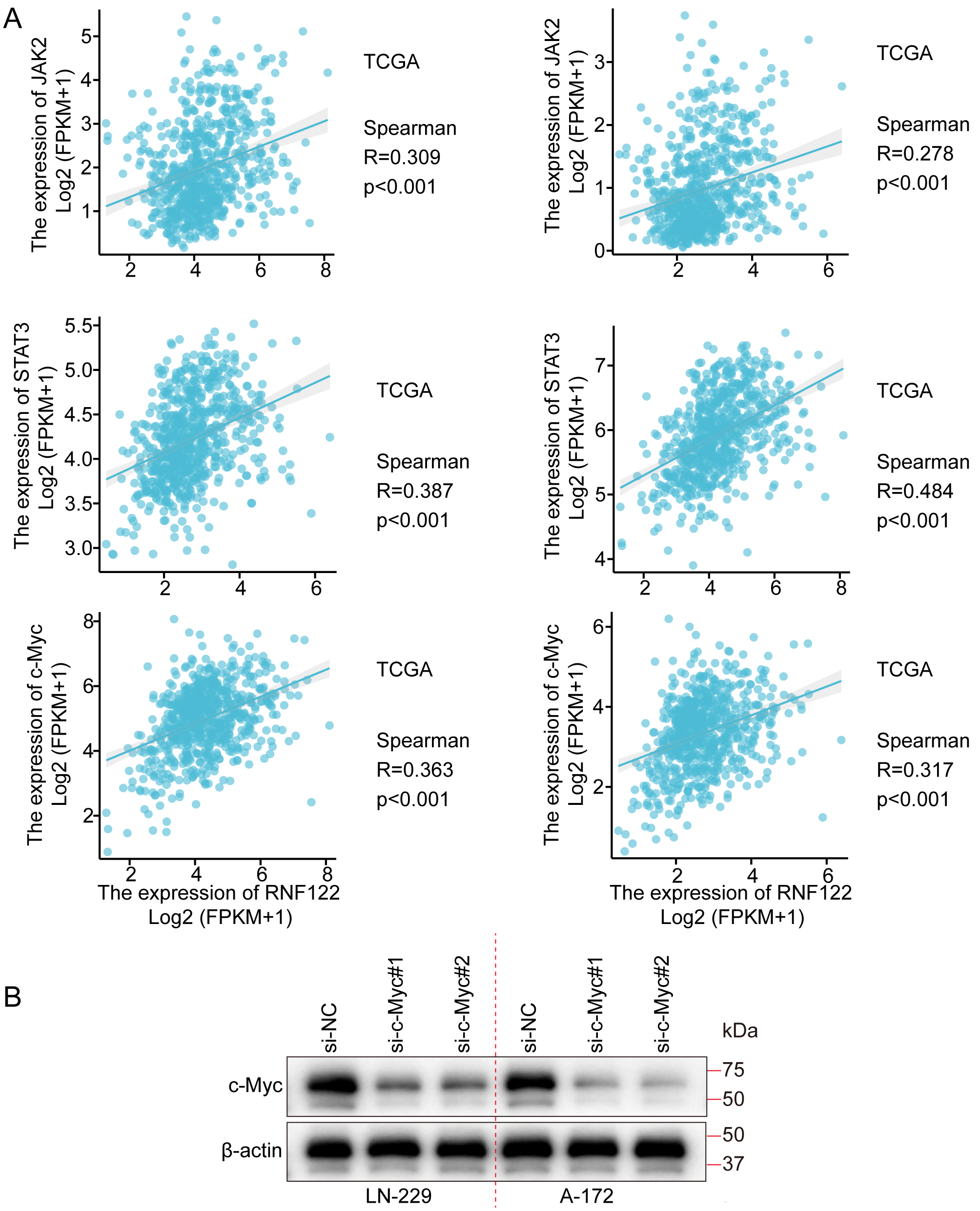


**Figure S4 A** TCGA database results show that RNF122 is positively correlated with the expression of JAK2/STAT3/c-Myc. **B** Validation of knockdown efficiency of c-Myc by WB. The means ± SDs are provided (n=3). ***P < 0.001 according to two-tailed Student t tests or one-way ANOVA followed by Dunnett tests for multiple comparisons.


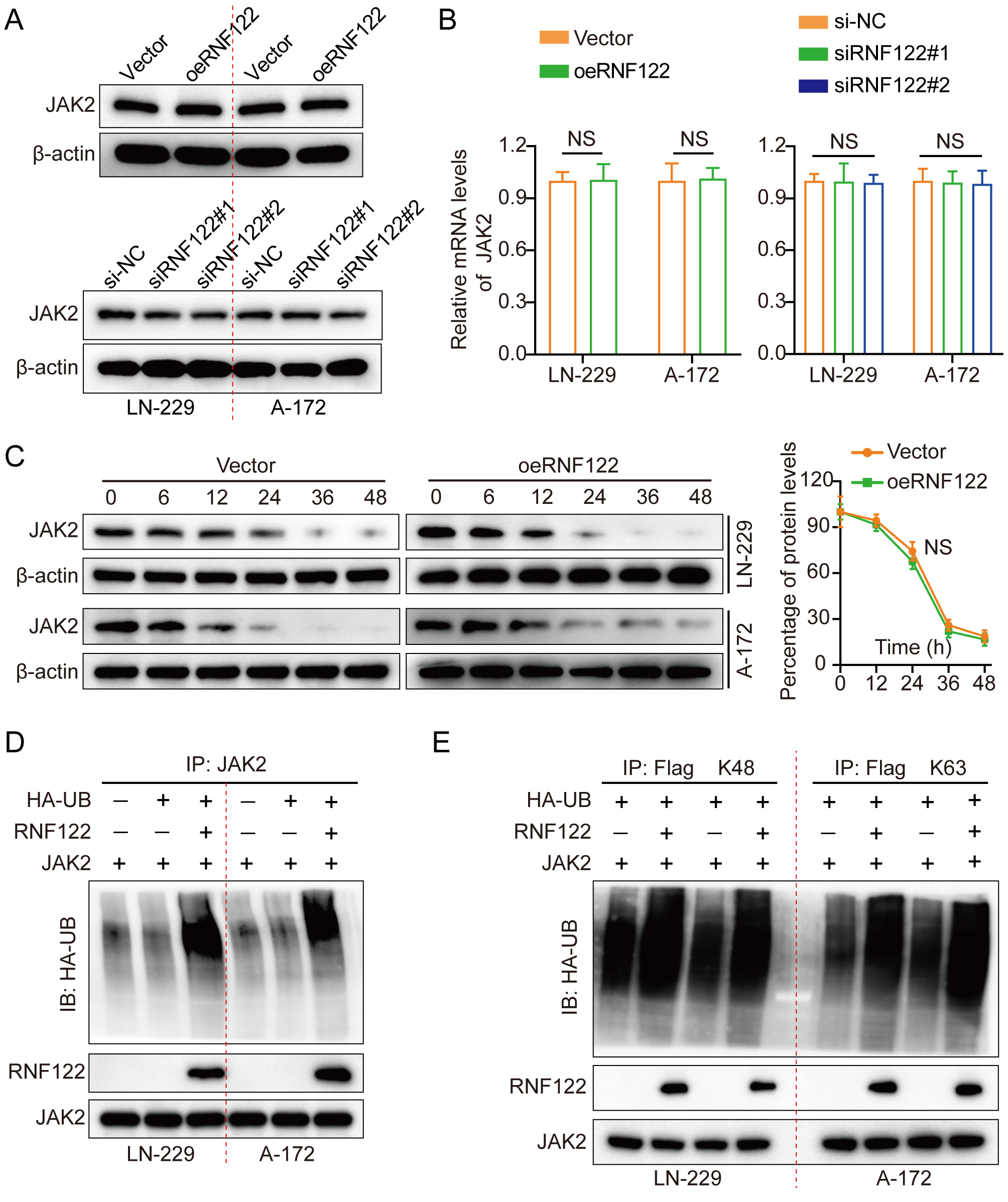


**Figure S5 Overexpression of RNF122 promotes K63-linked ubiquitination of JAK2.** RNF122 was overexpressed and knocked down in LN-229 and A-172 cells, respectively. WB (**A**) and qRT-PCR (**B**) experiments showed that RNF122 did not change the protein and transcript levels of JAK2. (**C**) Protein synthesis was inhibited by CHX, and cells were collected at different time points. Western Blot experiments showed that overexpression of RNF122 in LN-229 and A-172 cells did not accelerate the degradation of JAK2. (**D**) Overexpression of RNF122 in LN-229 and A-172 cells combined with Co-IP assay revealed that the ubiquitination level of JAK2 was increased. (**E**) Further identification of the type of ubiquitination modification of JAK2 revealed that RNF122 catalyzes K63-linked rather than K48-linked ubiquitination of JAK2. The means ± SDs are provided (n=3). ***P < 0.001 according to two-tailed Student t tests or one-way ANOVA followed by Dunnett tests for multiple comparisons.
