# Supplementary material for: RNF122 promotes glioblastoma growth via the JAK2/STAT3/c‐Myc signaling Axis
Source: CNS Neurosci Ther. 2024 Sep 1;30(9):e70017. doi: 10.1111/cns.70017 (PMC11366496; doi:10.1111/cns.70017)
Supplement: Supplementary file 1 — Data S1. [file CNS-30-e70017-s001.zip › cns70017-sup-0002-Supinfo02.pdf]

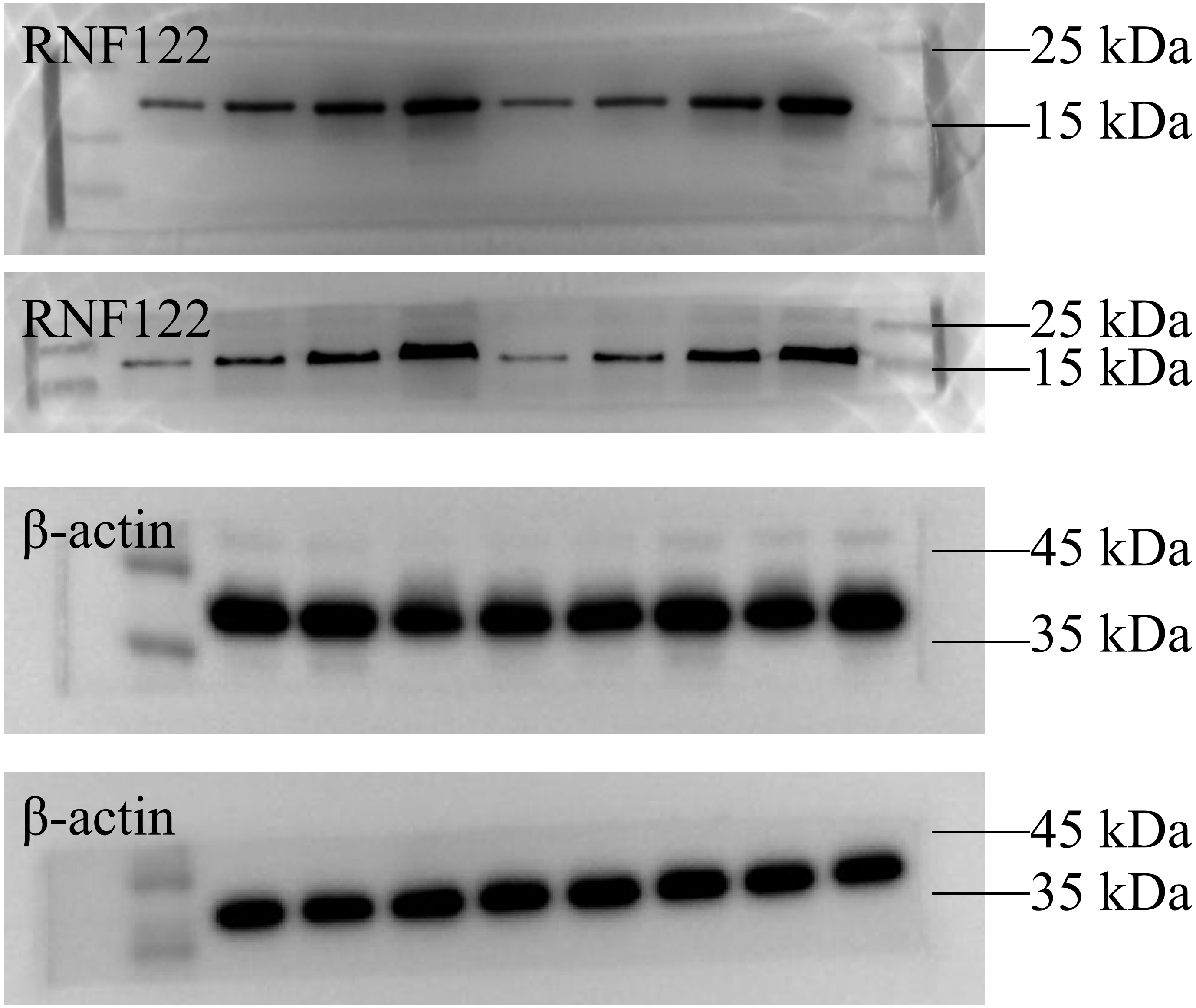

Full unedited gel/blot for Figure 1B

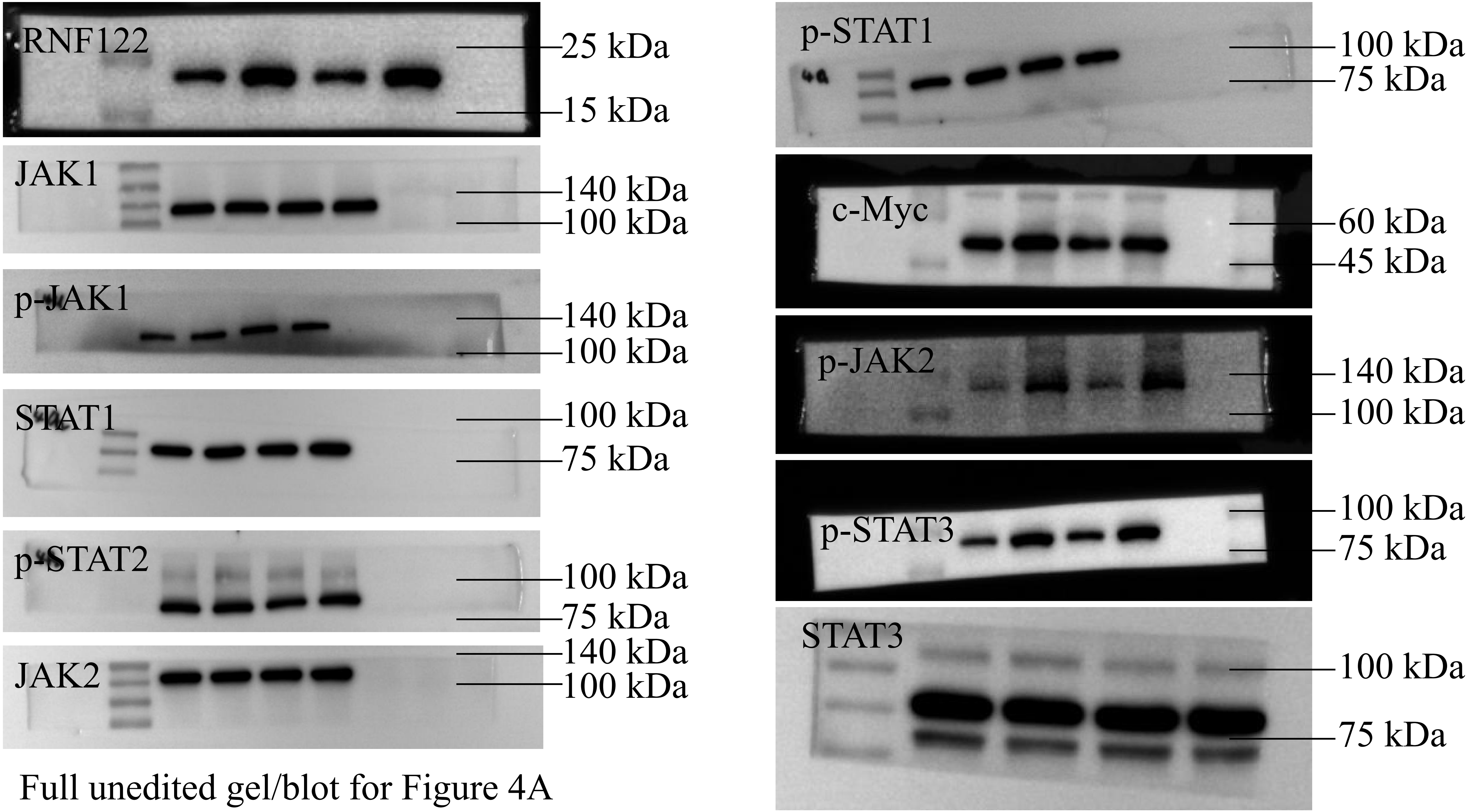

Full unedited gel/blot for Figure 4A

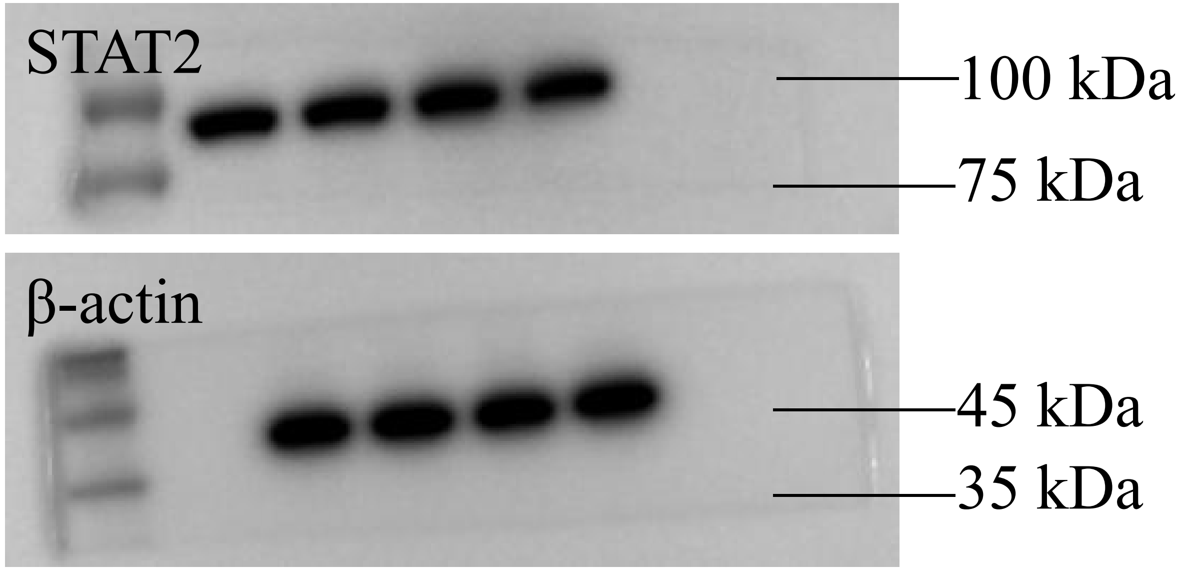

Full unedited gel/blot for Figure 4A

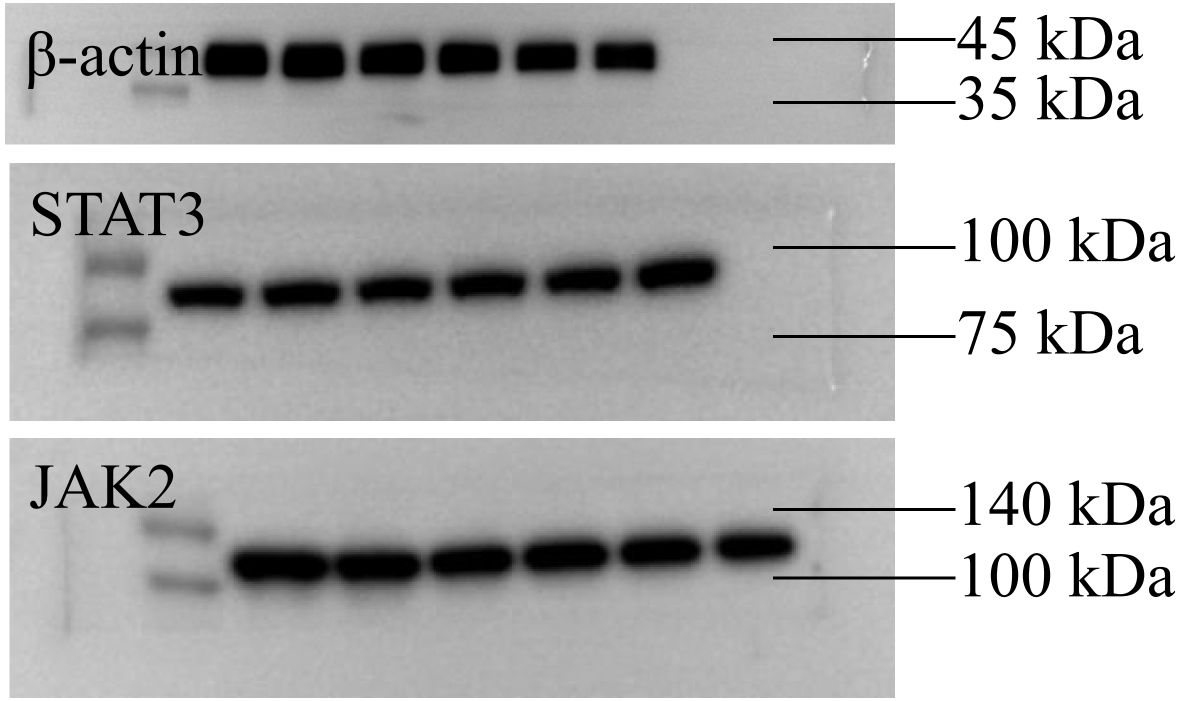

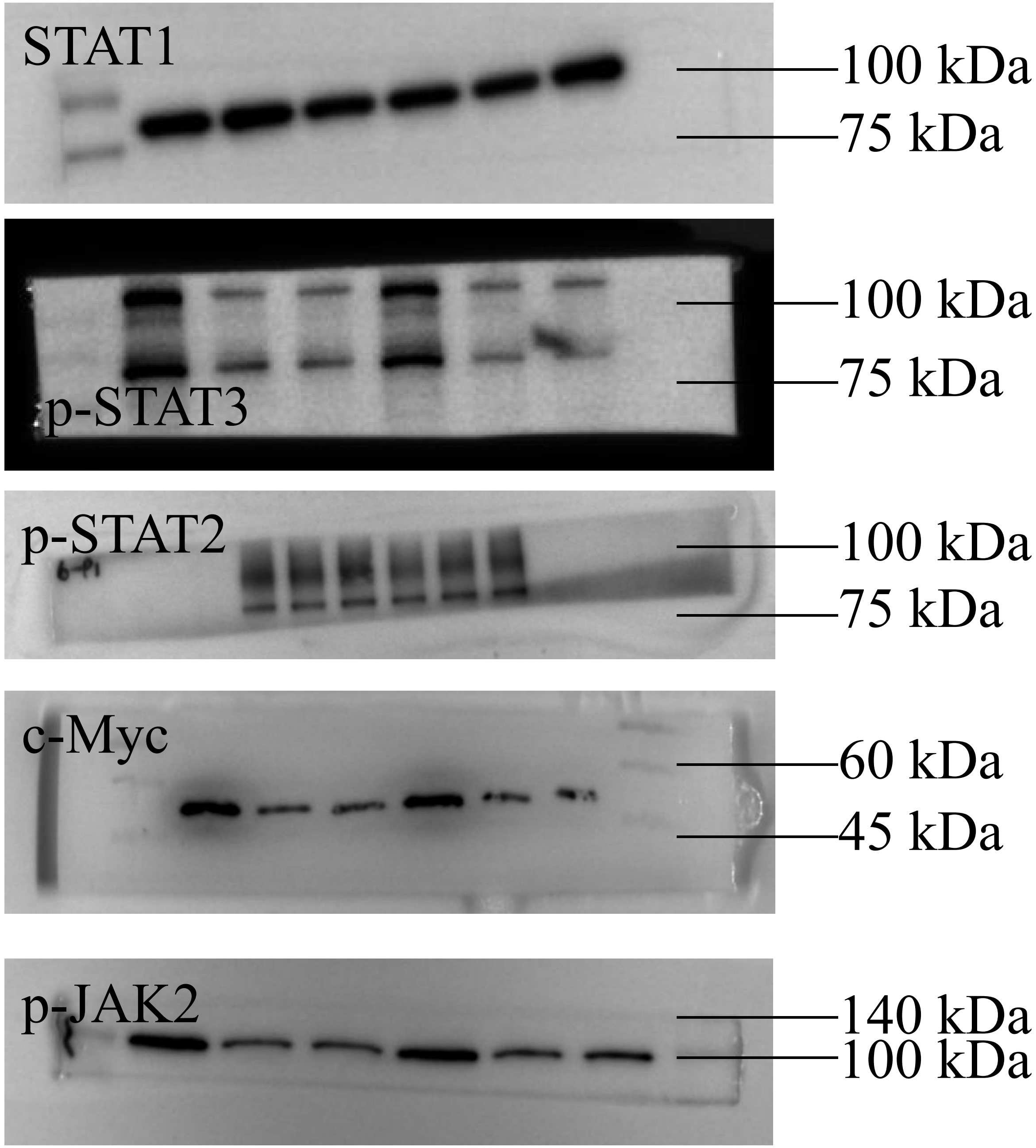

Full unedited gel/blot for Figure 4B

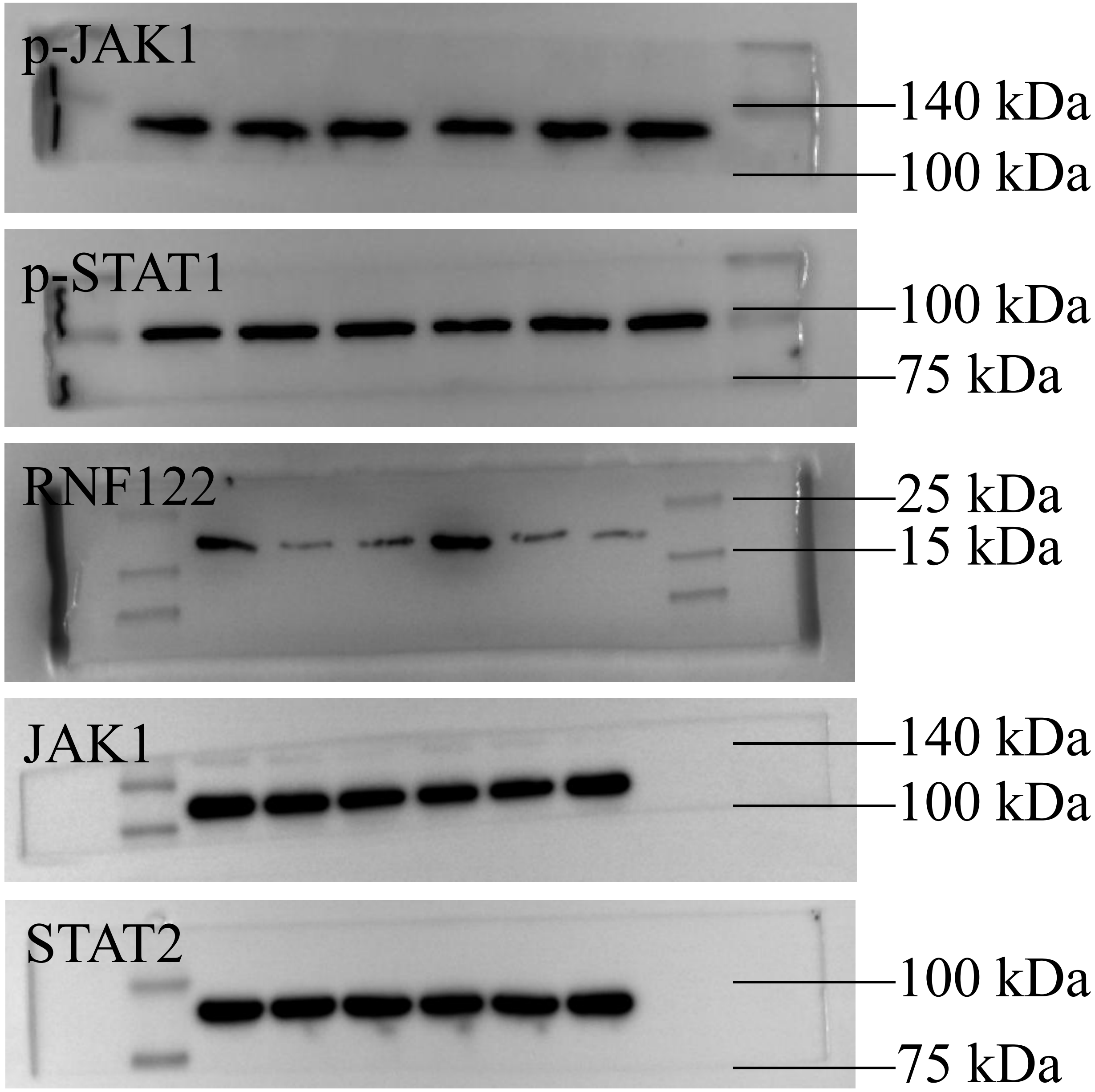

Full unedited gel/blot for Figure 4B

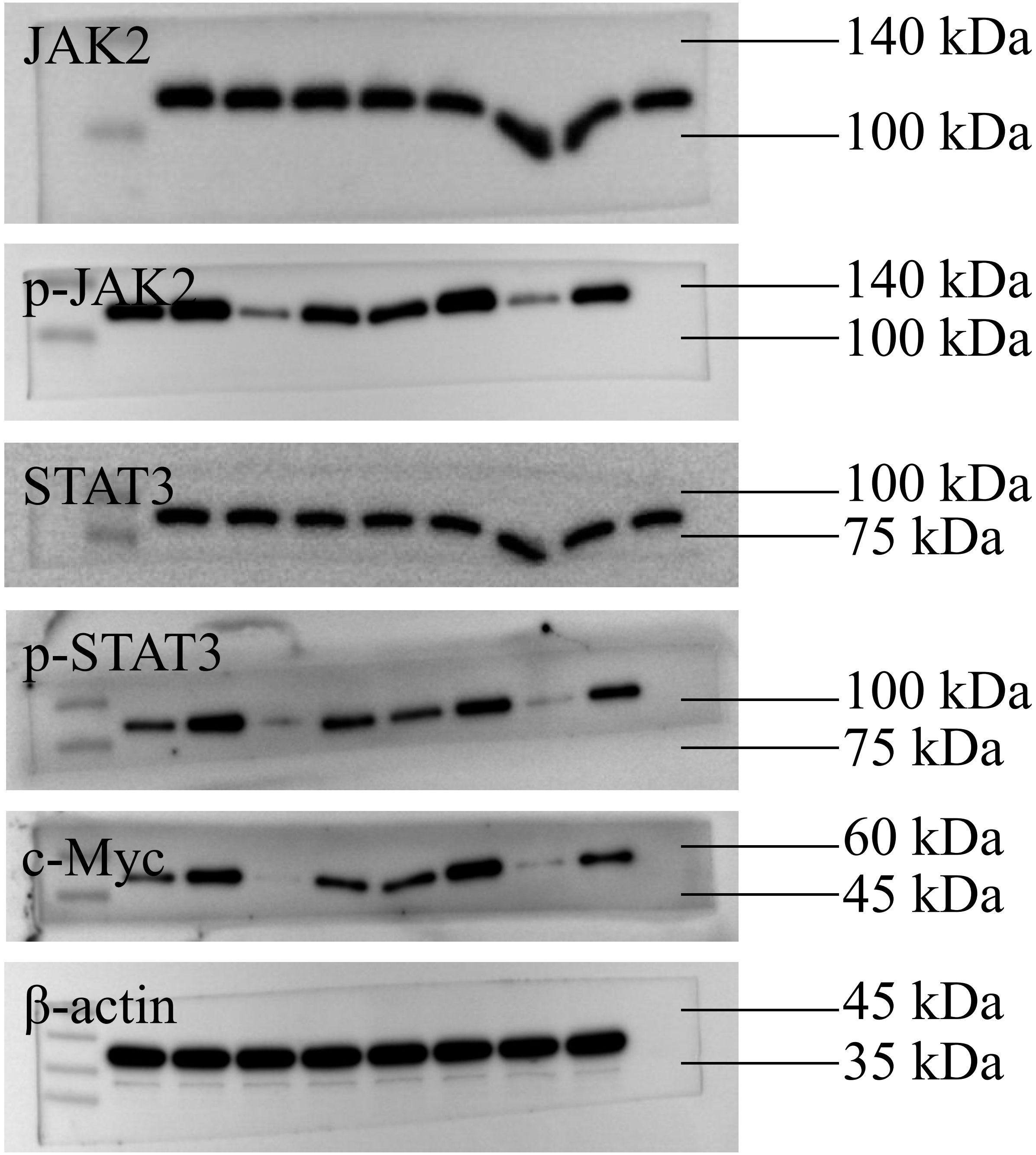

Full unedited gel/blot for Figure 5A

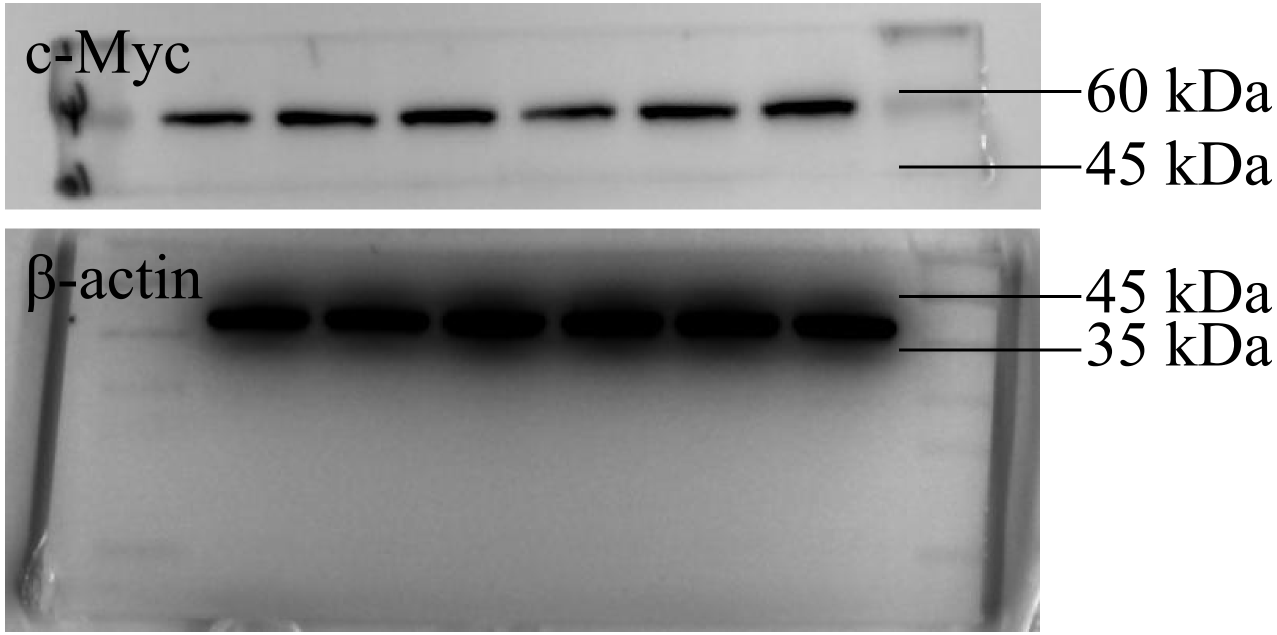

Full unedited gel/blot for Figure 7D

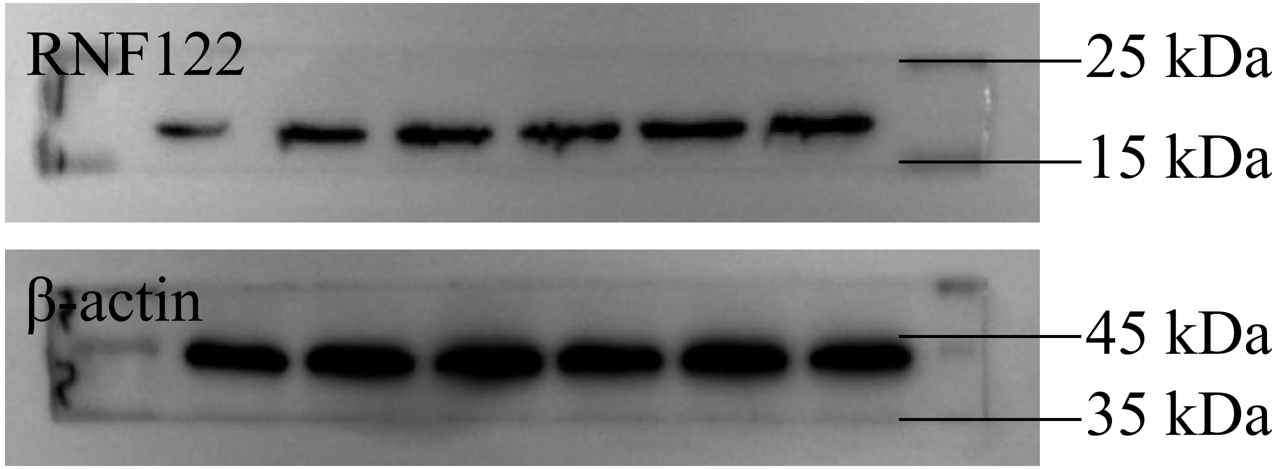

Full unedited gel/blot for Figure S1D

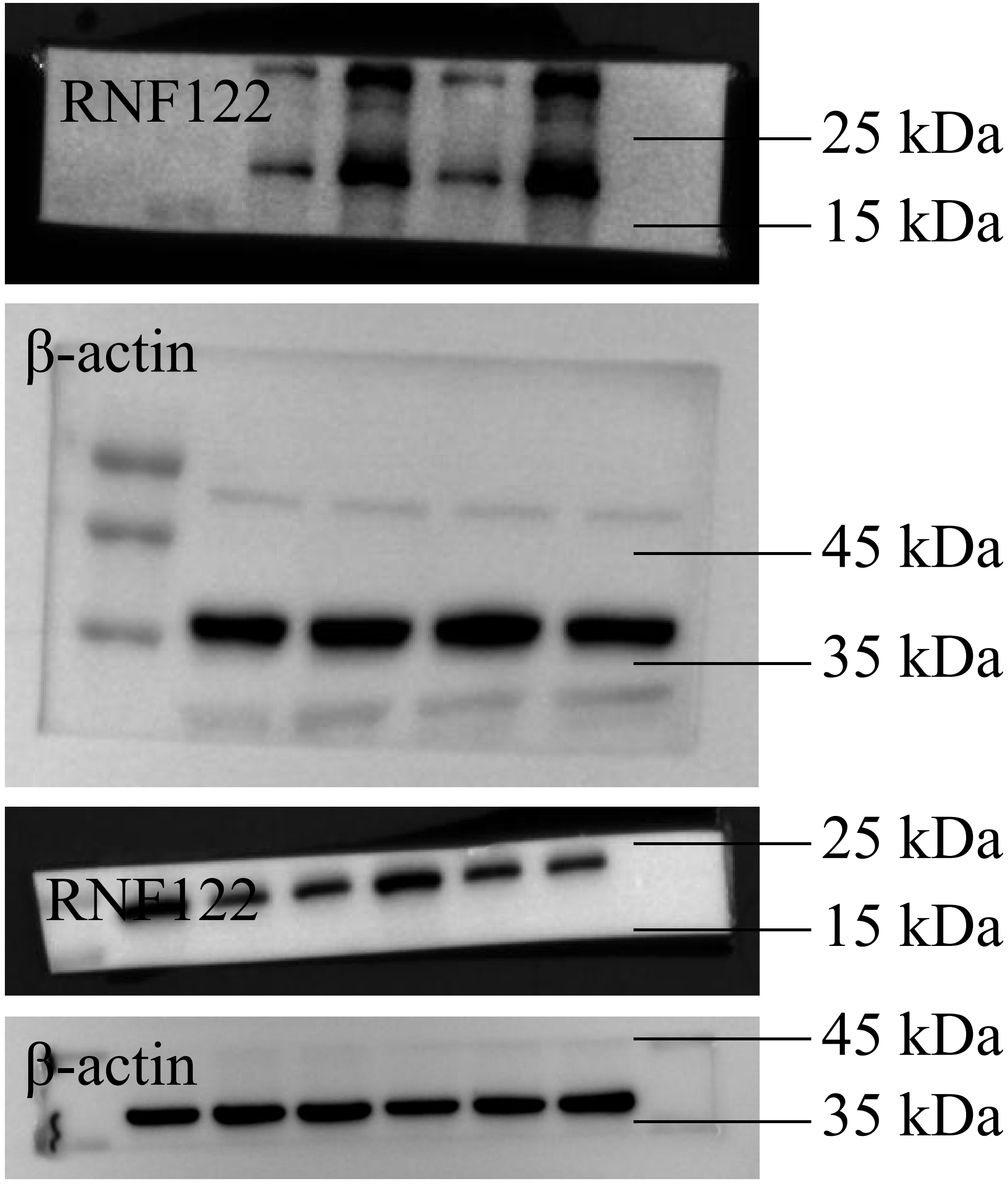

Full unedited gel/blot for Figure S1E

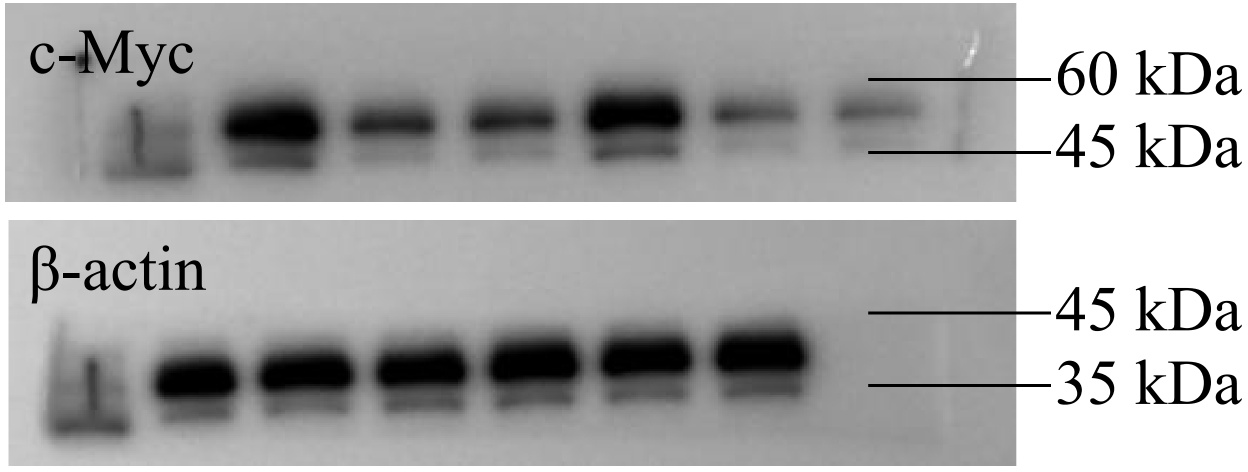

Full unedited gel/blot for Figure S4B

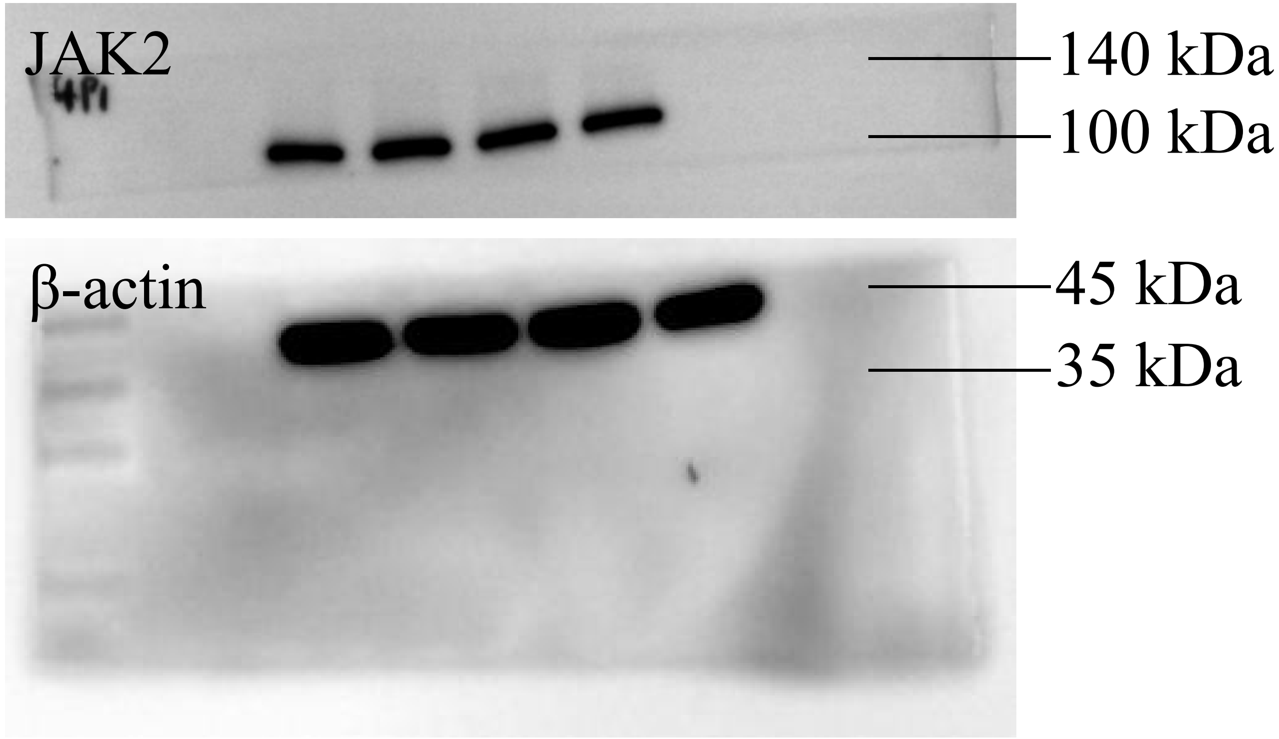

Full unedited gel/blot for Figure S5A

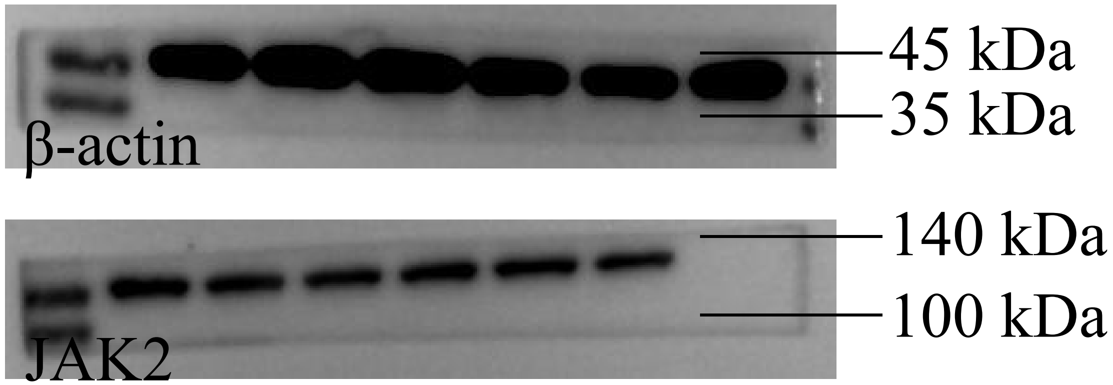

Full unedited gel/blot for Figure S5A

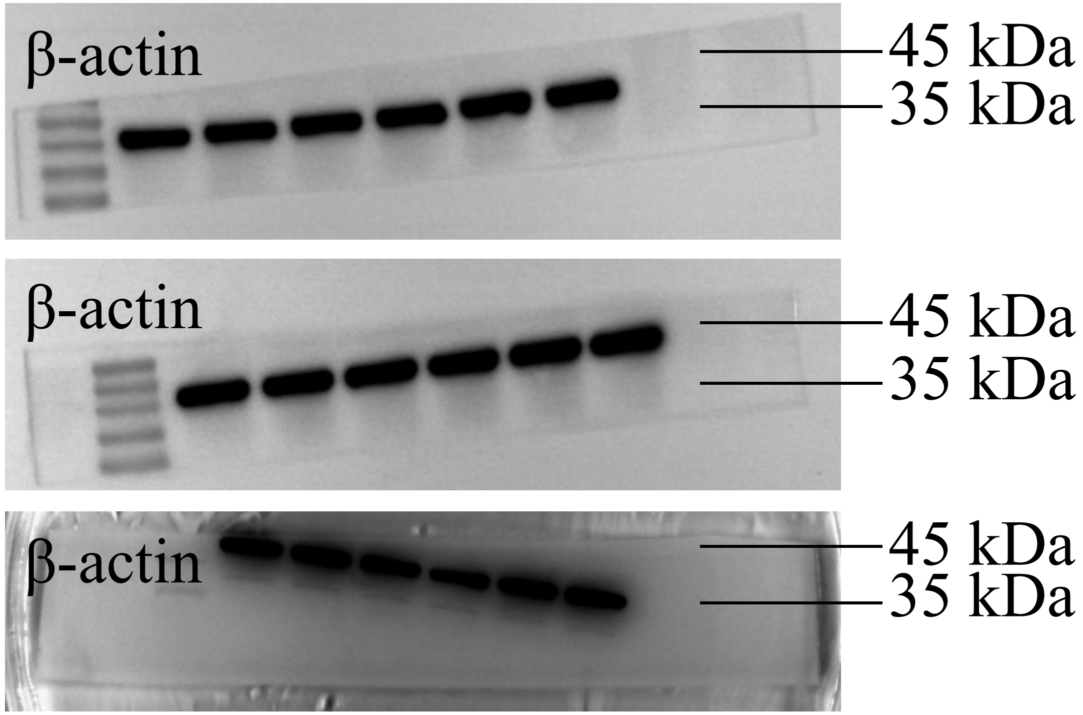

Full unedited gel/blot for Figure S5C

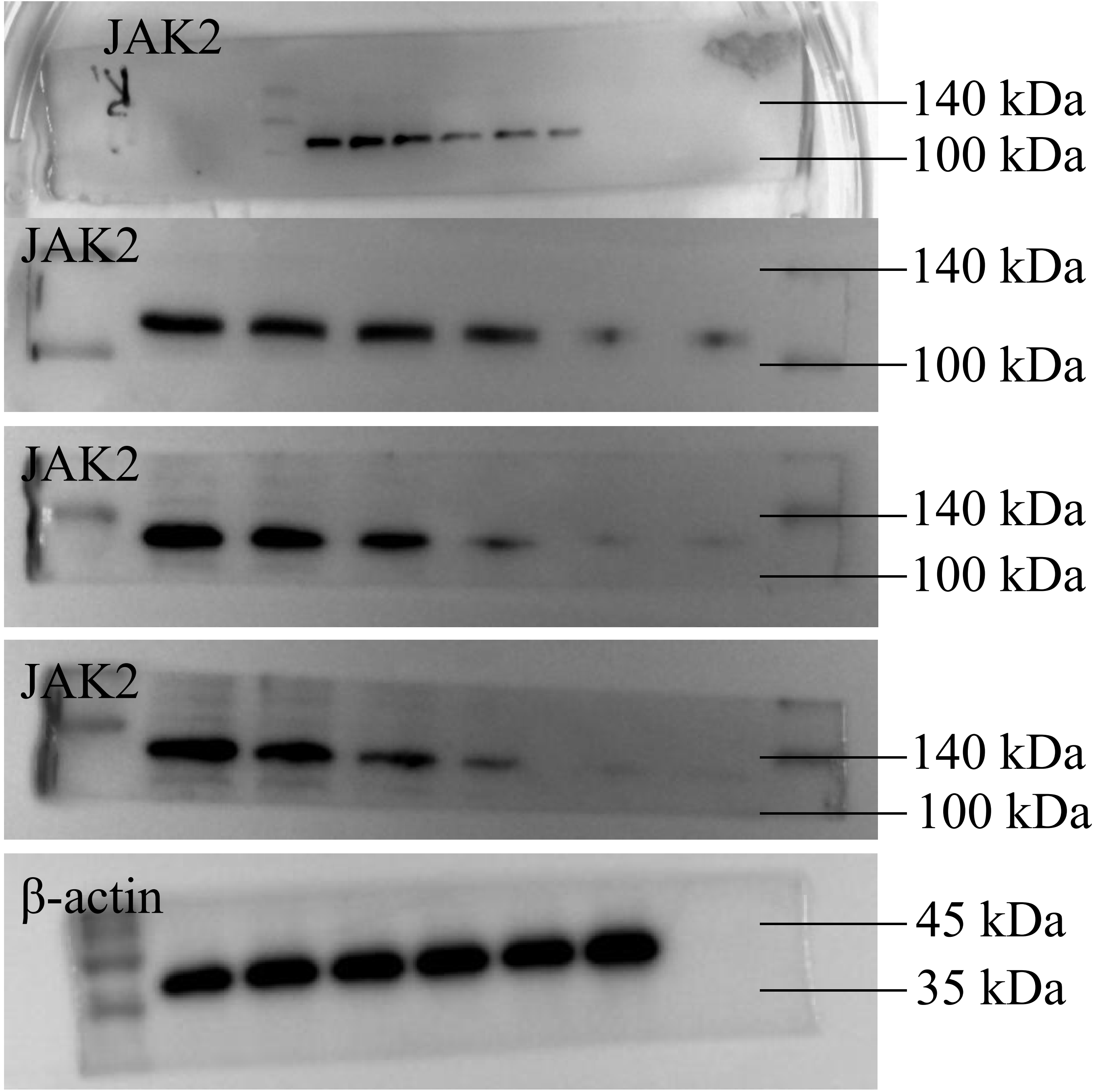

Full unedited gel/blot for Figure S5C

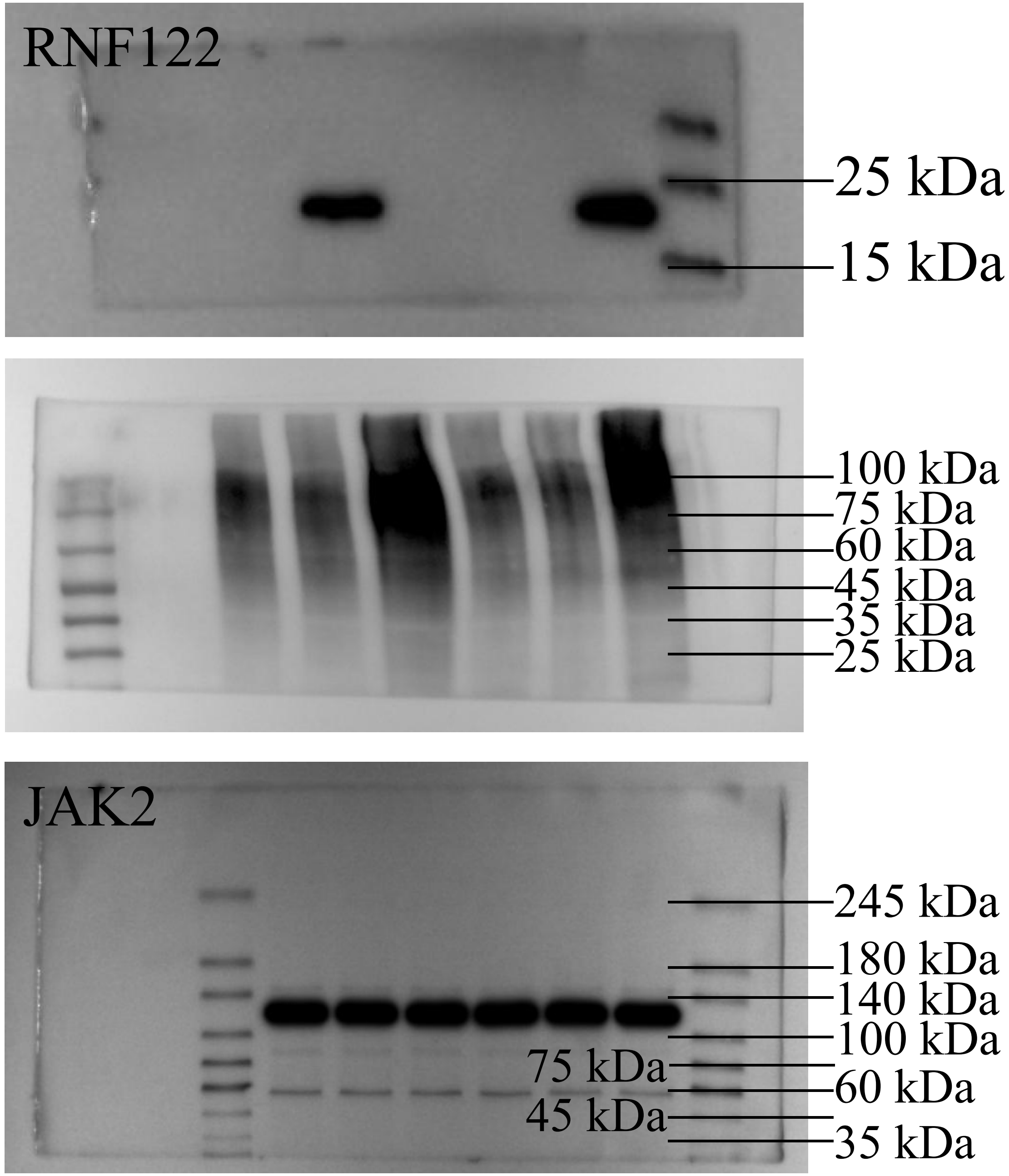

Full unedited gel/blot for Figure S5D

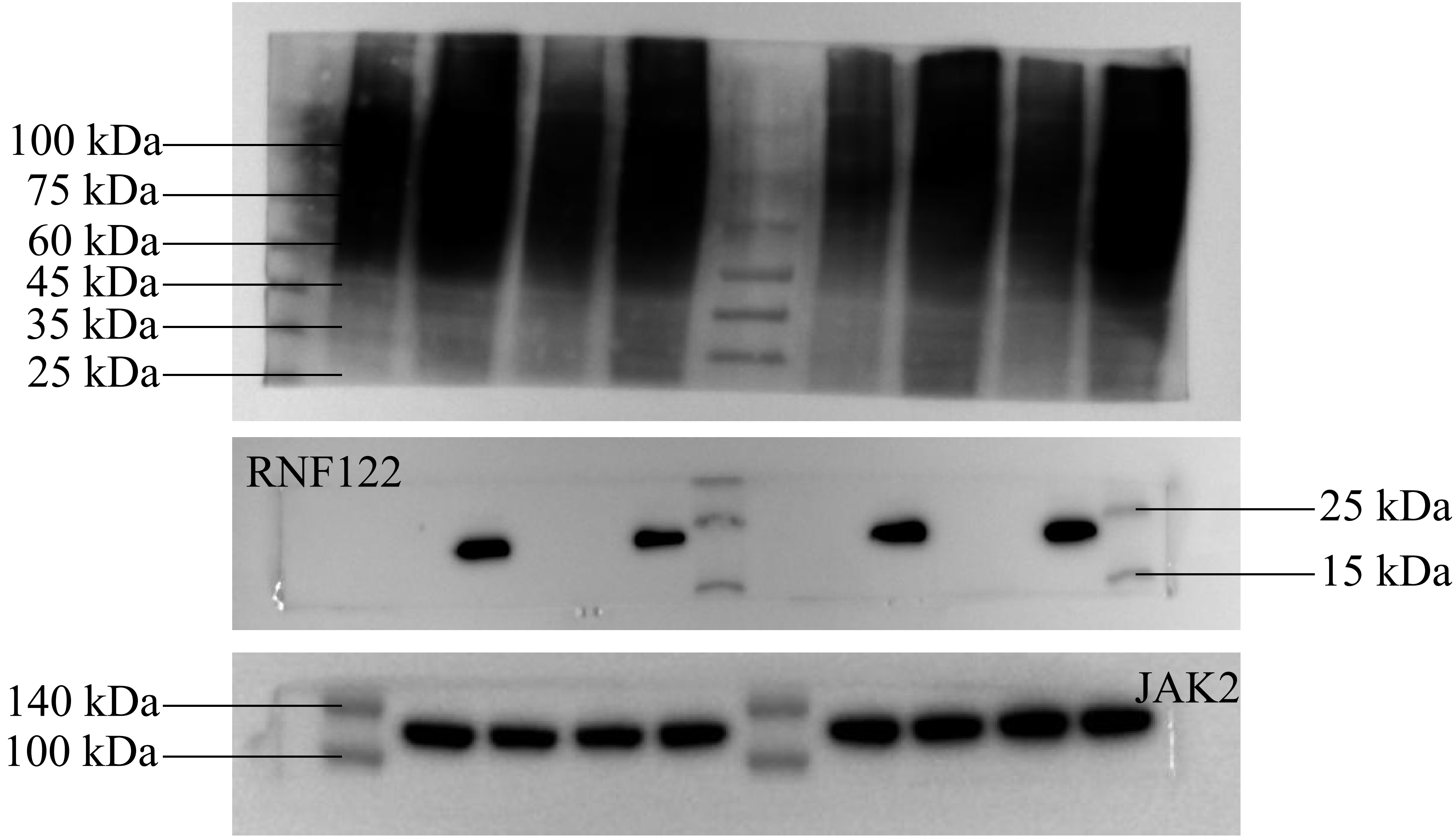

Full unedited gel/blot for Figure S5E
